# An evaluation based on the analytic hierarchy process and GGEbiplot on French fry potato genotypes in Yunnan, China

**DOI:** 10.3389/fpls.2023.1159848

**Published:** 2023-09-18

**Authors:** Shumin Liang, Wei Jiang, Yan Yang, Lili Lu, Jonathan L. Whitworth, Richard G. Novy, Lixian Bao, Ziyou Yin, Zhou Li, Pinggen He, Youxian Xu, Xianping Li

**Affiliations:** ^1^ Industrial Crops Research Institute, Potato Engineering Technology Research Center of Yunnan, Yunnan Academy of Agricultural Sciences, Kunming, Yunnan, China; ^2^ Aberdeen Research and Extension Center, United State Department of Agriculture-Agricultural Research Service, Aberdeen, ID, United States; ^3^ Zhaotong Academy of Agricultural Sciences, Zhaotong, Yunnan, China; ^4^ Lijiang Academy of Agricultural Sciences, Lijiang, Yunnan, China; ^5^ Agricultural Technology Extension Center of Xuanwei, Xuanwei, China

**Keywords:** potato, French fries quality, evaluation system, genotype-environment interactions, the analytic hierarchy process, Yunnan-China

## Abstract

A total of 33 potato (*Solanum tuberosum* L.) cultivars and breeding clones imported from the United States and two local cultivars (Yunshu 401 and Cooperation 88, CK) were planted and evaluated. To determine their suitability for processing into French fries at five locations (e1-e5) in Yunnan Province, China, we developed a comprehensive evaluation system using the analytical hierarchy process (AHP). Eleven evaluation indicators for French fry quality, yield, and agronomic characteristics with a relative importance (weight coefficients) of 0.483, 0.301 and 0.216, respectively, were used to analyze the 35 potato genotypes (designated g1-g35).The genotypes were ranked and the results revealed that (1) on the average, the 33 potato genotypes imported from the United States showed a lower performance compared to the local cultivars. Compared with the CK, they were classified as not vigorous (Mean 5.11 vs CK 7.75), matured earlier (Mean 5.79 vs CK 1.70), and had a low resistance to late blight (Mean 3735.59 vs CK 1418.55), requiring the use of fungicides to control the disease at the five trial locations. (2) The US cultivar ‘Defender’ (g3) ranked in the top six at all five test locations because it had higher yield (29.56 t h m^-2^), better fry quality (4.64), higher dry matter content (20.41%), better tuber length/width ratio (1.99), longer tubers (13.57cm), stronger plant vigor (7.17) and higher resistance to late blight (AUDPC: 3134.2). (3) By using GGEbiplot analysis, superior genotypes with high and stable yields were g3 and ‘Echo Russet’ (g33). ‘Yunshu 401’ (g34) and ‘Yukon Gem’ (g4) had high but not stable yields. The ideal test environments and hence experimental locations were Luquan (LQ, e2) and Lijiang (LJ, e4) which resulted in the best discrimination between genotypes among the five experimental locations in Yunnan. Overall, the developed evaluation system based on AHP and GGEbiplot analysis including 11 evaluation indicators for French fry quality, yield and agricultural traits can be a model for evaluation and promotion of new French fry cultivars, and evaluating and selecting the test location.

## 1 Introduction

China is the largest producer of potatoes (*Solanum tuberosum* L.) in the world with a production of 94.4 million tons in 2021, which accounted for 25.1% of the world’s total yield that year (FAO, 2023). Owing to increased urbanization and a preference for fast food, French fry is one of the primary modes of consumption of potato in China (Liu et al., 2017; Kaur et al., 2021). Frozen French fries are popular all over the world and sold in many supermarkets. In addition, the French fries in fast food restaurants are popular with children and adults.

Currently, only a few cultivars are suitable for processing into French fries in China due to the lack of potato germplasm resources, narrow genetic backgrounds and close genetic relationships (Xu and Jin, 2017). ‘Russet Burbank’ from the US and ‘Shepody’ from Canada are still the primary cultivars of raw potatoes to produce French fries in China. The import French fry potato genotypes from the US, particularly the Pacific Northwest, can greatly enrich the germplasm resources of potato in China (Jansky et al., 2009). However, the regions of the Pacific Northwest are generally arid, irrigated, and in high latitudes that are completely different from those in the low latitude plateau of Yunnan Province with adequate rainfall. It is unclear whether those imported potato cultivars are adapted to the growing environments in Yunnan.

Analytic hierarchy process (AHP) and GGE biplot can support the selection of suitable cultivars for Yunnan. AHP can evaluate germplasm resources (Wang et al., 2021; Zhao et al., 2022), screening new crop cultivars (Buciumeanu et al., 2021), and, therefore, be included in the decision-making of cultivars selection (Rahman and Saha, 2008; Ramírez-García et al., 2015). It uses weighting factors as input data in terms of their importance, ranks them and estimates the overall weight of individual criteria or parameter (Morandi et al., 2020; Singha et al., 2020; Yao et al., 2022). The GGE biplot combines the genotype effect (G) with genotype by environmental interaction effect (GE) and is widely used to integrate analysis of the various stability parameters to select stable and high-yielding germplasm resources in multiple environments (Yan et al., 2000; Ruswandi et al., 2022). Therefore, GGE biplot may overcome limitations of single indexes or tuber quality of French fries alone and indicate the overall advantages and disadvantages of potato genotypes (Abong et al., 2009; Waxman et al., 2018; Sawicka et al., 2021). However, there is no definition of the importance and the weight of evaluation indicators including lower and upper limits. Furthermore, there are also no standard field agronomic traits used to select potato cultivars for French fries processing. Thus it is urgent to select appropriate criteria and indicators for field agronomic traits and the quality of French fries.

The aim of this study was to construct a comprehensive evaluation system of potato genotypes to screen 33 imported and 2 local genotypes for French fry quality, yield and agricultural traits in order to agree on cultivars planting decisions in Yunnan. Furthermore, these traits were combined, and quality, yield and agronomic traits of the potato genotypes were evaluated by using AHP and GGEbiplot and results used to identify suitable cultivars for the Yunnan Province.

## 2 Materials and methods

### 2.1 Experimental genotypes

A total of 33 genotypes were imported from the US, and they were numbered in sequence as g1 to g33. In addition, ‘Yunshu 401’ (g34) and ‘Cooperation 88’ (g35,CK) were used as the local controls (**Supplementary Table 1**).

### 2.2 Experimental sites

The field experiments were conducted over a two-year period from May to October 2019 at three potato experimental stations: e1: Huize (HZ), e2: Luquan (LQ), and e3-1: Zhaotong (ZT), and from April to November 2020 at five potato experimental stations e1, e2, ZT (e3-2); and e4: Lijiang (LJ); and e5: Xuanwei (XW) in Yunnan Province, south western China (**Supplementary Figure 1**). The stations were typical low latitude, monsoon, and had vertical climatic (based on variable altitude) zones. The altitude of the spring potato planting area is generally above 1,900 m. The rainfall is generally between 250-650 mm, and the average temperature is generally approximately 15°C during the growth period. The geographic and meteorological data of the experimental sites were obtained from weather stations at the study sites (**Table 1**).

**Table 1:** Brief description of the geographic, metrological parameters of five experimental sites in Yunnan Province, southwest China.

Location	East longitude	North latitude	Altitude (m)	Average temperature in growing season	Cumulative precipitation during the growing season (mm)	annual precipitation(mm)	Frost-free period per year (days)	Sowing date	Harvesting date
e1(HZ)	103°19’12.0′′	25°53’24′′	2668	14.81	616.2	723	262	2019.5.8 2020.4.7	2019.9.24 2020.10.14
e2(LQ)	102°34’4′′	26°5’42′′	2794	–	–	–	–	2019.5.10 2020.4.1	2019.9.18 2020.10.23
e3-1(ZT)	103°38’42′′	27°16’35′′	1930	20.12	257	511.2	347	2019.5.10	2019.8.23
e3-2(ZT)	103°23’42′′	27°14’24′′	2480	14.39	621.6	753.2	292	2020.4.1	2020.9.14
e4 (LJ)	100°5’	26°47’	2780	14.68	636.2	664	211	2020.4.10	2020.11.3
e5 (XW)	102°6’33.24′′	26°13’13.39′′	2210	–	–	–	–	2020.4.8	2020.10.25

e1: Huize (HZ), e2: Luquan (LQ), e3-1: Zhaotong (ZT), e3-2: Zhaotong (ZT), e4: Lijiang (LJ), e5: Xuanwei (XW).

### 2.3 Experimental design

*In vitro* pathogen-free plantlets of the US cultivars and breeding clones were provided by the University of Idaho, Seed Potato Germplasm Program (Moscow, ID, USA), and each genotype was subsequently propagated to 800 plants in tissue culture at the Yunnan Academy of Agricultural Sciences (YAAS) Potato Center (Kunming,China) in May 2019. The plants derived from tissue culture were transplanted to the field at the three experimental stations (e1; e2; and e3-1) after the seedlings had been harden off for two weeks in greenhouse, the day temperature was 22°C and the night temperature was 18°C. After transplanting, the plants derived from tissue culture were irrigated by sprinklers every day, and irrigated 10 days. There were no any other treatments and plant protection applied at seedling stage. The plants were spaced 0.3 m, and the rows were spaced 0.7 m. A randomized complete block (RCB) design was used with three replicates in 2019-2020. Each plot was planted with 80 plants derived from tissue culture of each genotype with three replicates at the three experimental stations in 2019. The other plants derived from tissue culture were transplanted to sterile substrate in a greenhouse in Song Ming, Yunnan, and the mini-tubers that were harvested from greenhouse in the 2019 were used as seed potatoes for the experiment in 2020. Each plot was planted with 60 plants of every genotype with three replicates at the five potato experimental stations in 2020. Late blight (*Phytophthora infestans*) was controlled in all the locations for all genotypes, but late blight was controlled by chemicals in XW, the late blight still broke out. After the plants died completely (because of late blight), the tubers were harvested by hand in both years.

### 2.4 Data collection

#### 2.4.1 Data of quality traits

##### 2.4.1.1 Evaluation of French fry quality

Ten of the most uniform and least blemished potato tubers were selected from each of the three replicated plots after the harvest at maturity. The soil and debris were removed from the tubers, which were then washed. They were hand-peeled starting at the stem-end (basal) side of all 10 potato tubers using stainless steel knives, and the peeled tubers were cut along the long axis with an automatic French fry cutter machine into 7 mm × 7 mm rectangular strips; the central four strips of each tuber were collected (Waxman et al., 2018). The 40 central strips were then soaked, blanched, dried, and par-fried (**Supplementary Figure 2**). Palm oil was used to fry the potato strips. The French fries of all genotypes were handled and processed with the same method (**Supplementary Figure 2**). The crispness, exterior shell, mealiness, moistness, texture variation and internal appearance of French fries of each genotype were evaluated by three panelists from the Yunnan Academy of Agricultural Sciences. The panelists were asked to score the six parameters using a standard rating scale from 1 to 9 as previously described. For example, for Crispness, 1 means less crisp and 9 means severely undesirable crunchy; for exterior shell: 1 means weak, 9 means tough; for all six parameters, 5 indicates the most optimal quality (**Supplementary Table 6**) (Waxman et al., 2018; Liang et al., 2020).

##### 2.4.1.2 The dry matter content

More than 5 kg tubers of each plot was sampled to determine the content of dry matter during the harvest. The air and water weight of each material were measured using an electronic portable scale (1 g), and the specific gravity was calculated using Equation (1). The dry matter content was calculated using Equation (2), which is the regression equation between the dry matter content and the specific gravity as found in the Mepkep table (Heltoft et al., 2017).


(1) T=(A-30)/[(A-30)-B]……

(2) D=213.9*T-211.3……

Where T is the specific gravity; A is the weight in air, and B is the weight in water. D is the dry matter content, and the weight is provided in g.

##### 2.4.1.3 Tuber length, width and the length/width

The longest and widest positions of 20 potato tubers were measured with a digital Vernier caliper, and the measured data were automatically recorded in a computer. The length/width = the tuber length/tuber width.

#### 2.4.2 Yield and its components

**Total yield:** At harvest, all the tubers in each plot were collected. The yield, tuber count, and grading were performed manually. The tubers were harvested by hand and graded on weight (large tubers > 100 g, medium tubers 50–100 g, and small tubers< 50 g). The tubers > 100 g were considered to be commercial-grade tubers. The number of single tubers in each grade was counted and weighed to determine the total yield. The ratio of large tubers was determined as the percentage of tubers that weighed >100 g relative to the total yield of fresh tubers. The weight per marketable tubers was determined as the weight of tubers that weighed > 100 g/the number of the tubers that weighed > 100 g. The ratio of small tuber was determined as the percentage of tubers that weighed< 50 g relative to the total yield of fresh tubers.

#### 2.4.3 Agronomic properties

The plant vigor was determined by examination after emergence (emergence rate > 80%) and again after 30 days (**Supplementary Table 2**).

The plant maturation was investigated from middle to late August (**Supplementary Table 3**).

Resistance to late blight was assessed by the area under the disease progress curve (AUDPC). From the first susceptible plant was found, then the incidence of late blight was evaluated in every 7 days for three to eight times. The AUDPC was calculated based on the incidence of late blight. The method of International Potato Center (Forbes et al., 2014) was used as the standard to investigate the rate of incidence and calculate the AUDPC.

### 2.5 Statistical analysis

#### 2.5.1 Data analysis

The data of the experiments were collected in 2019 and 2020, respectively. And the data used for analysis by AHP and GGE was the average of two years at e1 and e2, the average of e3-1 in 2019 and e3-2 in 2020, and the data at e4 and e5 in 2020. Because *in vitro* plantlets and the microtubers were virus-free, and there was no difference in growth, disease resistance, length/width of tubers (**Supplementary Figure 3**) in 2 years, the performance of the 33 cultivars and breeding clones at 3 experimental locations in two years was consistent. Therefore, the differences between 2019 and 2020 and the differences between 3 and 5 experimental locations were not analyzed.

#### 2.5.2 The data dimensionless according to the positive and negative effects of a single factor

The raw data, such as the “crispness value” obtained from the panelists, ranged between 1and 9. However, since 5 were the best score of six parameters, and 9 and 1 were the worst scores, it was impossible to directly evaluate the parameters and make strategic decision. In addition, other data such as the value of “small tuber ratio (< 50 g)” and the late blight AUDPC have negative effects on the yield and income of growers. Therefore, the raw data were calculated using an “S-type” curve model, an inverse “anti-S-type” curve model or the parabolic model (ladder type), and the formulas used were Function (1) to Function (7) (**Table 2**). In formula (1): f(x): membership function; x: measured data; a: low limit; b: high limit; b1 and b2: the best score corresponding to the measured data/the highest limit. This was also used to describe functions (2) to (7). The scores of the 11 indicators (see 2.5.3, sub-criteria P1-P11) were added as the score of genotypes at the five sites.

**Table 2:** S-type and anti-S-type membership functions, parameters, and weights.

Index The x of the Function(1-7)		Membership function	Membership function parameter (Critical value)				Weight	
			a(Lower limit)	b1	b2	b(Up limit)		
Quality of french fries	Crispness	Function(1)	1	5	5	9	0.149	The weight of quality traits 0.483
	The hardness of exterior shell		1	5	5	9		
	Taste fineness		1	5	5	9		
	Moistness		1	5	5	9		
	Texture variation		1	5	5	9		
	Internal appearance		1	5	5	9		
Dry matter content (%)			19.5	25	25	100	0.123	
Length/width of tubers		Function(2)	1.50	1.75	1.75	2.5	0.123	
Length of tubers (cm)		Function(3)	5				0.088	
The ratio of big tuber			0			100	0.107	The weight of yield traits 0.301
The weight per marketable tubers (g/one tuber)		Function(4)	0	149.3	294.1		0.077	
Small tuber ratio (width<5cm)		Function(5)	0	12	12	100	0.064	
Yield (Kg/hm^2^)		Function(3)	0			100	0.053	
Maturity		Function(6)	1			9	0.089	The weight of agronomic traits 0.216
Plant vigor		Function(1)	1	5	5	9	0.074	
Late blight resistance (AUDPC)		Function(7)	0			100	0.053	

Effect of index for tuber economic and quality benefit. Quality of french fries: Parobolic,5 is the highest score,100; Length/width of tubers:Parobolic, the value=1.75 is the highest score, 100, if the value is less than 1.5, the score is 10; Dry matter content (%): The value is less than 19.5%, the score is10, the value is 25%,the score is100, the value is more than 25%, the score decreases; Length of tubers (cm): The value<5cm,the score is 10, the value >5cm, the score is the value/the max value of all cultivars; The ratio of big tuber: The max value of all cultivars,the score is100, the score of others is the value/the max value of all cultivars; The weight per marketable tubers (g/one tuber): The value is in the 0-149.3, the score is the value/149.3, the value is in the 149.3-294.1, the score is 100; Small tuber ratio (width<5cm): The value is 0, the score is 100, the value is 12%, the score is 10, the value is in the 12-100%, the score is 10-0; Yield (Kg/hm^2^): The max value of all cultivars,the score is100, the score of others is the value/the max value of all cultivars; Maturity: The value is in the 1-9, the score is 100-10; Plant vigor: Parobolic,5 is the highest score,100; Late blight resistance (AUDPC): The max value of all cultivars, the score is10, the score of others is calculated by Function(7).

Membership function (1):


$$\begin{array}{l} f(x)=\left\{ \begin{array}{l} 10,x\leq a\\ 0.1+0.9*\left(\frac{x-a}{b1-1}\right),a<x<b1\\ 100,x=b1,b1=b2\\ 1-0.9*\left(\frac{x-b2}{b-b2}\right),b2<x>b \end{array}\right. \end{array}$$

Membership function (2): 


$$f(x)=\left\{ \begin{array}{l} 10,x\leq 19.5\\ \frac{x}{25},19.5<x<25\\ 100,x=25\\ 1-\left(\frac{x-25}{25}\right),25<x<100 \end{array}\right.$$

Membership function (3):


$$f(x)=\left\{ \begin{array}{l} 0,x\leq a\\ \frac{x}{\max(x)},x>a \end{array}\right.$$

Membership function (4):


$$f(x)=\left\{ \begin{array}{l} 0,x\leq 0\\ \frac{x}{149.3},0<x<149.3\\ 100,149.3<x<294.1 \end{array}\right.$$

Membership function (5):


$$f(x)=\left\{ \begin{array}{l} 100,x\leq a\\ 1-\frac{x}{12},0<x<12\\ 10,x=12\\ 0.1*\frac{\max(x)-x}{\max(x)},12<x<100 \end{array}\right.$$

Membership function (6): 


$$f(x)=\left\{ \begin{array}{l} 10,x\leq 1\\ 1-\frac{x}{9},0<x<9 \end{array}\right.$$

Membership function (7):


$$f(x)=\left\{ \begin{array}{l} 10,x=\max(x)\\ 1-\left(\frac{\frac{x}{\max(x)}-0.2}{1-0.2}\right),x>0 \end{array}\right.$$

#### 2.5.3 Establishment of a comprehensive evaluation system by AHP

According to the interrelationship and subordinate levels of the factors that are directly related to the selection of high-yield and high-quality potato cultivars imported from the US, different levels of aggregation and combination were conducted to build a hierarchical framework model (**Table 3**).

**Table 3:** Research framework for potato genotype suitability modeling in Yunnan Province, China.

Goal hierarchy(A)	Criteria: First hierarchy (B)	Sub–criteria: Second hierarchy (P)
Suitability of potato genotypes in Yunnan, China	The quality traits (B1)	Quality of French fries (P1), Dry matter content (P2), Length/width of tubers (P3), Length of tubers (P4)
	The yield traits(B2)	The ratio of large tuber(P5), The average weight per marketable tuber (P6), Small tuber ratio(width<5cm) (P7), Yield(P8)
	The growth traits(B3)	Maturity (P9), Plant vigor (P10), Late blight resistance (P11)

Goal hierarchy (A) was a comprehensive evaluation of the imported potato processing cultivars from the US in Yunnan, China. The first hierarchy (B) was composed of three primary criteria (the quality traits [B1], yield traits [B2] and agronomic traits [B3]). The second hierarchy (P) was composed of 11 sub-criteria (Quality of French fries [P1], Dry matter content [P2], Length/width of tubers [P3], Length of tubers [P4], The ratio of large tubers [P5], The weight per marketable tuber [P6], Small tuber ratio [P7], Yield [P8], Maturity [P9], Plant vigor [P10], and Late blight resistance [P11]). The scores of the comprehensive evaluation system were used to select the French fry processing genotypes that were high-quality, high-yielding, vigorous and moderately resistant to late blight.

The importance of each criterion and the ranking of criteria were based on the scoring of experts in potato breeding. Further, the method used an underlying proportion criterion recording scale to rate the relative preference on a one-to-one basis of each criterion (**Table 3**). In the first hierarchy, the quality traits (B1) were significantly more important than the yield traits (B2), and B2 was extremely important compared with the growth traits (B3). The second hierarchy in the B1, the quality of French fries (P1), was significantly more important than the dry matter content (P2) and further importance ranking was P2=P3>P4. In the second hierarchy in the B2, importance of the ratio of large tubers was P5>P6>P7>P8. In the second hierarchy in the B3, importance of maturity was P9> P10 >P11.

In the construction of the judgment matrix, the weights of factors were calculated from the judgment matrix, the maximum eigenvalue of matrix (λ max), and the corresponding eigenvectors were obtained by calculation and checked for consistency. The judgment of consistency could be checked by the CR (Consistency ratio) of CI (Consistency index) with the appropriate value (**Supplementary Tables 4**, **5**), and the four CR were 0.031, 0.008, 0.008 and 0.015, respectively (**Table 4**), and the fact that the CR values were all< 0.1 is a reasonable level of consistency (Malczewski, 1999; Kamble and Raut, 2019). So, the weight coefficients based on the relative importance, and proportion of various evaluation indicators of a certain tested object were 0.149(P1), 0.123(P2), 0.123(P3), 0.088(P4), 0.107(P5), 0.077(P6), 0.064(P7), 0.053(P8), 0.089(P9), 0.074(P10), 0.053(P11) respectively. Therefore, the weight coefficients of quality traits was 0.483(P1, P2, P3 and P4), the weight coefficients of yield traits was 0.301(P5, P6, P7 and P8), and the weight coefficients of agronomic traits was 0.216(P9, P10 and P11).

**Table 4:** Judgement matrix and the results of a consistency check.

A-B					B1-Pi					
A	B1	B2	B3	W	B1	P1	P2	P3	P4	W
B1	1	1.4	2.52	0.484	P1	1	1.4	1.4	1.68	0.307
B2	1/1.4	1	1.8	0.301	P2	1/1.4	1	1	1.2	0.255
B3	1/2.52	1/1.8	1	0.215	P3	1/1.4	1	1	1.2	0.255
λmax=3.032 CI=0.016 RI=0.52					P4	1/1.68	1/1.2	1/1.2	1	0.182
CR=0.031<0.1					λmax=4.024 CI=0.008 RI=0.89 CR=0.009<0.1					
B3-Pi					B2-Pi					
B3	P9	P10	P11	W	B2	P5	P6	P7	P8	W
P9	1	1.4	1.68	0.412	P5	1	1.2	1.44	2.016	0.356
P10	1/1.4	1	1.2	0.343	P6	1/1.2	1	1.2	1.68	0.255
P11	1/1.68	1/1.2	1	0.245	P7	1/1.44	1/1.2	1	1.4	0.212
λmax=3.016 CI=0.008 RI=0.52					P8	1/2.016	1/1.68	1/1.4	1	0.177
CR=0.015<0.1					λmax=4.024 CI=0.008 RI=0.89 CR=0.009<0.1					

The abbreviation of CR, CI and RI mean consistency ratio, consistency index and random index.

### 2.6 GGEbiplot was used to analyze the interaction between genotypes and the environment

To determine the influence degree of GE (Genotype × Environment interaction) on stability and adaptability of exotic potato genotypes, the 33 US genotypes and breeding clones were planted in five different ecological zones in Yunnan Province, China. The ecological zones or environments were evaluated using the repeatable part in GE to determine which zones were suitable for genotype screenings. Genotype evaluation, test environment evaluation and discriminating ability of the environments were analyzed using a GGEbiplot. The comparison and histogram of all 11 indicators were analyzed using R software (Vienna, Austria). All the raw data were sorted and calculated by Microsoft Excel 2010 (Redmond, WA, USA).

## 3 Results

### 3.1 Quality traits, yield traits and growth traits of the studied potato genotypes

#### 3.1.1 Quality traits

**Figure 1:**
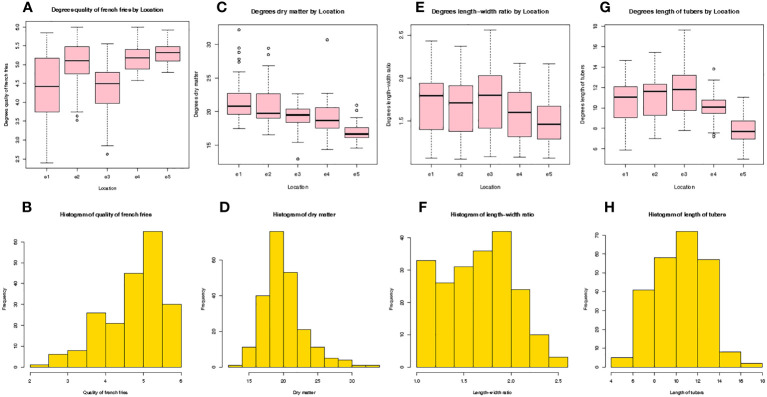
A comparison and histogram of the quality of French fries **(A, B)**, dry matter **(C, D)**, length to width ratio **(E, F)**, and length of tubers **(G, H)** averaged across five sites in the spring in Yunnan Province, China. (HZ: e1; LQ: e2; ZT: Average of e3-1and e3-2; LJ: e4; XW: e5).

Quality traits were used to evaluate the fitness of tubers for French fries processing from 5 experimental stations. The average value of the quality of French fries was 5.0-5.5 at e5 > e4 > e2. The average value of quality of French fries was closest to 5.0 at e2, and the average value of quality of French fries was 4.0-5.0 at e3 > e1. Simultaneously, e3 had the lowest recorded score between 2.5 and 3.3 (**Figure 1A**). The highest frequency histogram of the average value of quality of French fries was 4.5-5.5 (**Figure 1B**). The average value of dry matter content was 16-22% (**Figure 1C**). The potatoes grown at e1 had the largest average dry matter content, and the average dry matter content was ordered as e1 > e2 > e3 > e4 > e5. The lowest average dry matter content was at e5. The largest dry matter content > 30% and was recorded at e1 and e4, while the lowest dry matter content was recorded at e3. The highest frequency histogram of the dry matter content was between 18% and 22% (**Figure 1D**).The average value of the length/width of the tubers was 1.5-1.8 (**Figure 1E**), and the largest average length/width of the tubers was at e3 (**Figure 1E**). The average length/width of the tubers was ordered as e3 > e1 > e2 >e4 > e5. The tubers had the lowest length/width at e5, which was nearly 1.5, and the largest length/width of tubers > 2.5 at e3. The highest frequency histogram of the length/width of tubers was 1.5-2.0 (**Figure 1F**). The average value of length of tubers was 7-12 cm (**Figure 1G**), and the largest average length of tubers was at e1, which was nearly 12 cm, and the average length of tubers was ordered as e3 > e2 > e1 > e4 > e5. The tubers at e5 were the shortest at nearly 8 cm. The highest frequency histogram of the length of tubers was 8-14 cm (**Figure 1H**). In summary, the performance of the tuber quality traits were not consistent at the five experimental stations, and the average value of quality of French fries was highest in e5, the highest average dry matter content was in e1,and the highest average value of the length/width of the tubers and length of tubers were in e3.

#### 3.1.2 Yield traits

**Figure 2:**
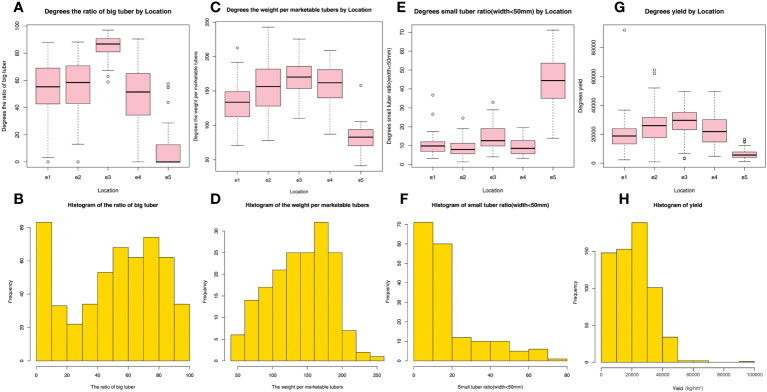
A comparison and histogram of the ratio of large tubers **(A, B)**, the weight per marketable tuber **(C, D)**, ratio of small tubers (width< 5 cm) **(E, F)** and yield **(G, H)** at five sites in the spring in Yunnan Province, China.

The yield components such as the ratio of large tubers and the small tuber ratio directly affect the yield of potatoes and their economic income per unit area. Our results showed that the highest average value of the ratio of large tubers was found at e3 (**Figure 2A**), and the average ratio of large tubers was ordered as e3 > e2 > e1 > e4 > e5. The highest frequency histogram of the ratio of large tubers was between 50% and 88% except for e5 (**Figure 2B**). The highest average value of the weight per marketable tubers was at e3 (**Figure 2C**), and the average weight per marketable tubers was ordered as e3 >e4 > e2 > e1 > e5. The highest frequency histogram of the weight per marketable tubers was between 120 g and 200 g (**Figure 2D**). The highest average value of the small tuber ratio (44.8%) was observed at e5 (**Figure 2E**), and the average value of small tuber ratio in the other four sites was approximately 10%. The average small tuber ratio was ordered as e5 (44.8%) > e3 (15.1%) > e1 (11.0%) > e4 (9.3%) > e2 (8.8%). The highest frequency histogram of small tuber ratio was between 0 and 20% (**Figure 2F**). The highest average value of yield (kg/hm^2^) was at e3 (**Figure 2G**), and the average yield was ordered as e3 > e2 > e4 > e1 > e5. The highest frequency histogram of the weight per marketable tuber was 15,000-30,000 (kg/hm^2^) (**Figure 2H**). A yield higher than 90,000 (kg/hm^2^) was recorded at e1, and the yield recorded at e2 was more than 60,000 (kg/hm^2^), and there was a lowest yield record at e3. In summary, the ratio of large tubers and the weight per marketable tubers were higher, the yield was higher, but the lower small tuber ratio the higher yield such as in e3.

#### 3.1.3 Agronomic traits

**Figure 3:**
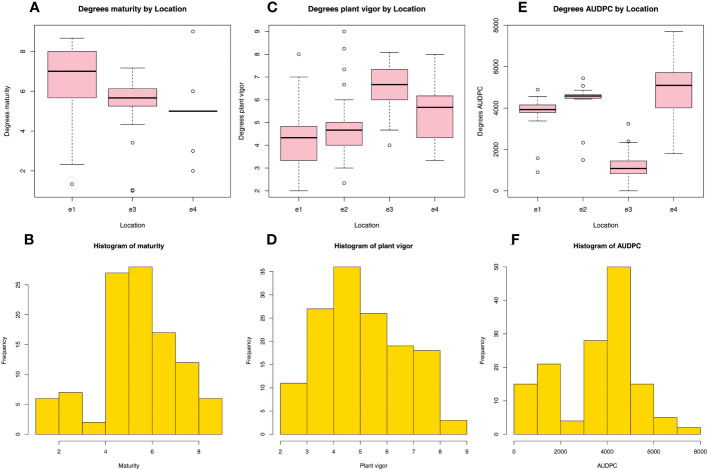
A comparison and histogram of the maturity **(A, B)**, plant vigor **(C, D)** and AUDPC **(E, F)** of five sites in the spring in Yunnan Province, China. AUDPC, area under the disease progress curve.

Agronomic traits (maturity, plant vigor and AUDPC) were always used to analyze and evaluate the disease resistance and genetic diversity of imported cultivars. Our results showed that: the average value of maturity was close to 7 at e1 (**Figure 3A**), while it was close to 6 at e3 and 5 at e4. The highest frequency histogram of maturity was 4-6 (**Figure 3B**). However, the value of maturity data of e2 and e5 were missing. The average value of plant vigor was close to 7 at e3 (**Figure 3C**), 6 at e4, close to 5 at e2 and close to 4.5 at e1. The highest frequency histogram of plant vigor was 4-5 (**Figure 3D**). However, the value of plant vigor data of e5 was missing. The highest average value of the AUDPC was at e4 (**Figure 3E**), and the average AUDPC was ordered as e4 > e2 > e1 > e3. The highest frequency histogram of AUDPC was 4,000-5,000 (**Figure 3F**). However, the value of AUDPC data of e5 was missing. In summary, the good performance of agronomic traits indicates that these cultivars were with strong adaptability at e3.

### 3.2 Evaluation of each genotype in five sites using AHP

We calculated the selected 11 indicators based on the constructed judgment matrix in AHP, and obtained the scores of the cultivars at each location. By constructing a pairwise comparison judgment matrix to determine the important of different factors on screening excellent potato cultivars, errors caused by accidental factors were avoided. The cultivars with the highest score in five sites were as follows: g1 (‘Premier Russet’) in e2 and e3, g31 (‘AO03123-2’) in e1, g34 (‘Yunshu401’) in e4 and e5, respectively (**Supplementary Tables 7**–**11**). The second ranked cultivars in the five sites were as follows: g34 in e1, g3 (‘Defender’) in e4, g7 (‘Alpine Russet’) in e2, g2 (‘Palisade Russet’) in e3 and g10 (‘Castle Russet’) in e5. The third ranked genotypes in the five sites were as follows: g3 in e1, g2 in e4, g32 (‘AO96305-3’) in e2, g21 (‘A03921-2’) in e3 and g3 in e5. In summary, g3 was in the top six genotypes in all five locations indicating that it appeared the most suitable for Yunnan, China, of the US entries.

### 3.3 GGEbiplot evaluation results from 33 US potato genotypes and control cultivars Yunshu 401 and Cooperation 88

#### 3.3.1 Selection of the most desirable genotype

**Figure 4:**
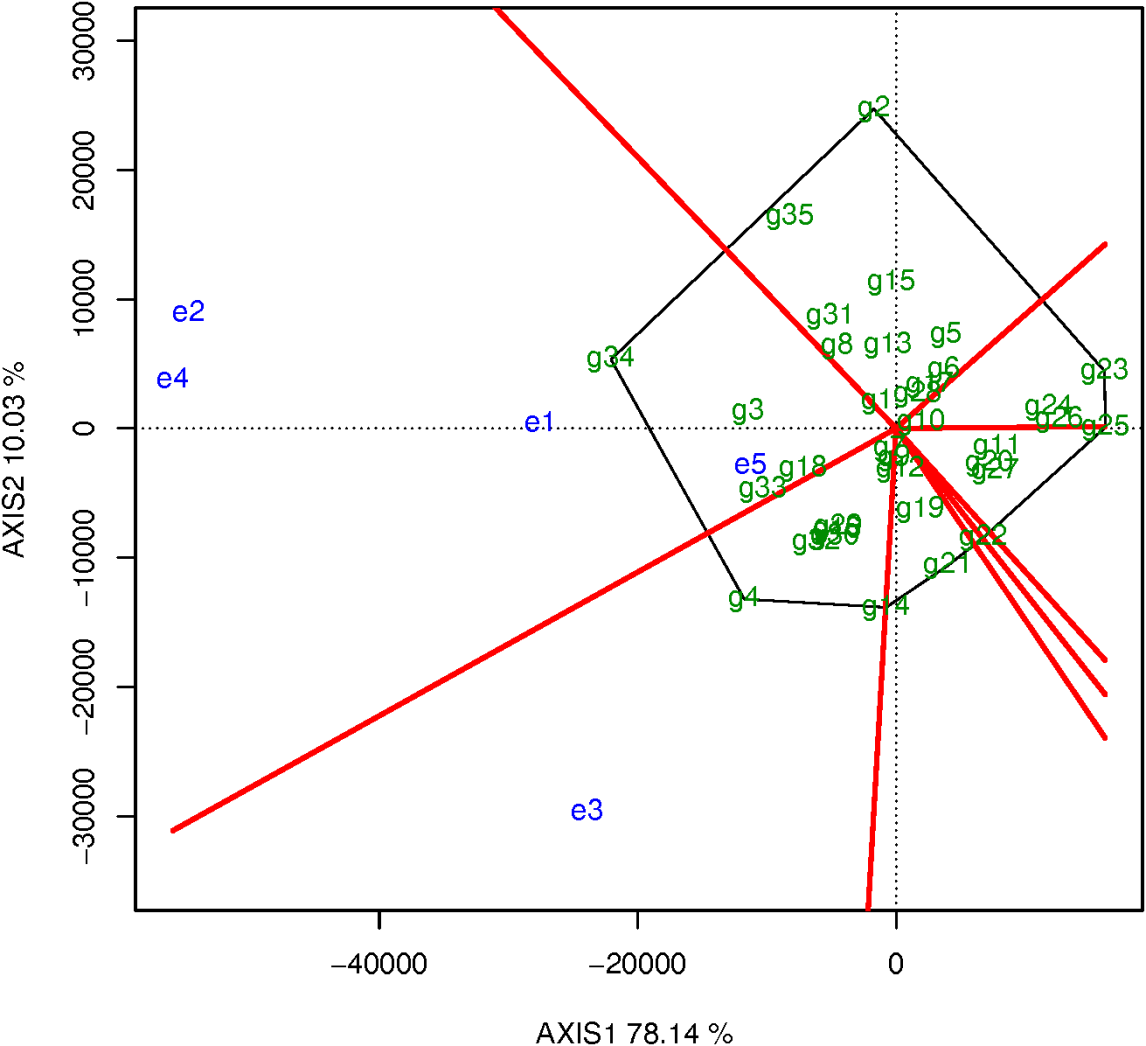
Superior genotypes based on the evaluation.

GGE Biplot is used to select cultivars with good comprehensive characters, strong adaptability, stability and high yield. The variation of 78.14% of the G and the interaction between GE can be effectively explained using a GGEbiplot through analyzing our 5 experimental locations data (**Figure 4**). The four test locations of e1, e2, e4 and e5 were considered as one group, and e3 was another group. The genotype g34 (‘Yunshu401’) which located at the top corner of the polygon was determined to be the most suitable genotype with the highest yield at these four test sites, followed by g3 (‘Defender’). Since g34 was the control genotype, g3 was technically the superior genotype with the highest yield at these four sites. In addition, g4 (‘Yukon Gem’) located at the top corner of the polygon in another group was also the superior genotype with the highest yield at e3 (**Figure 4**). the genotypes g2, g23, g25 and g14 were located at the other top corner of the polygon but the traits were not desirable. Over all, g3 and g4 were the most adaptable genotypes in Yunnan.

#### 3.3.2 The relationships among environment and the ability to discriminate their degree of representation

**Figure 5:**
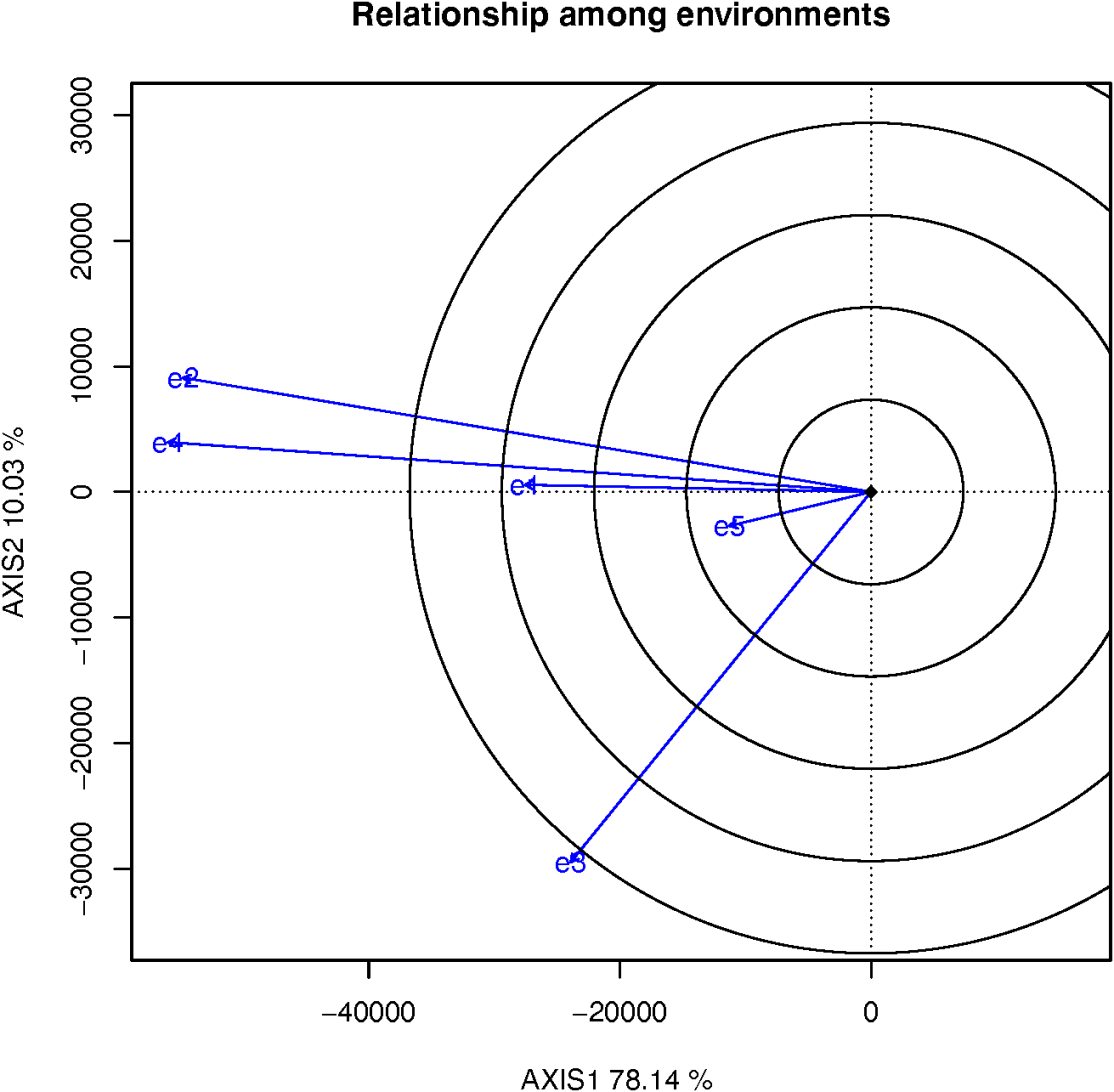
The relationship among environment.

The cosine of angle between the two environmental vectors approximates the genetic correlation coefficient between them. If the angle less than 90° indicates a positive correlation, and the angle greater than 90° indicates a negative correlation, and the angle close to 90° indicates no correlation. Positive correlation indicates that the two environments have similar ranking of cultivars; negative correlation indicates that the two environments have a different ranking of cultivars, and the included angles of these five test sites were all< 90° (**Figure 5**). This showed that there was a close positive correlation between these five test sites. The order of genotypes was similar, and the close positive correlation indicates that some test sites may be repeatedly established. The length of the environment vector is the degree to which the experimental location is able to distinguish varieties. The environmental vectors of e2, e4 and e3 were longer than those of the e1 and e5 test sites, and the e2, e4 and e3 test sites could clearly discriminate between the genotypes (**Figure 5**). Therefore, the e1 and e5 test sites can be removed to reduce the expense of these tests without affecting the evaluation.

**Figure 6:**
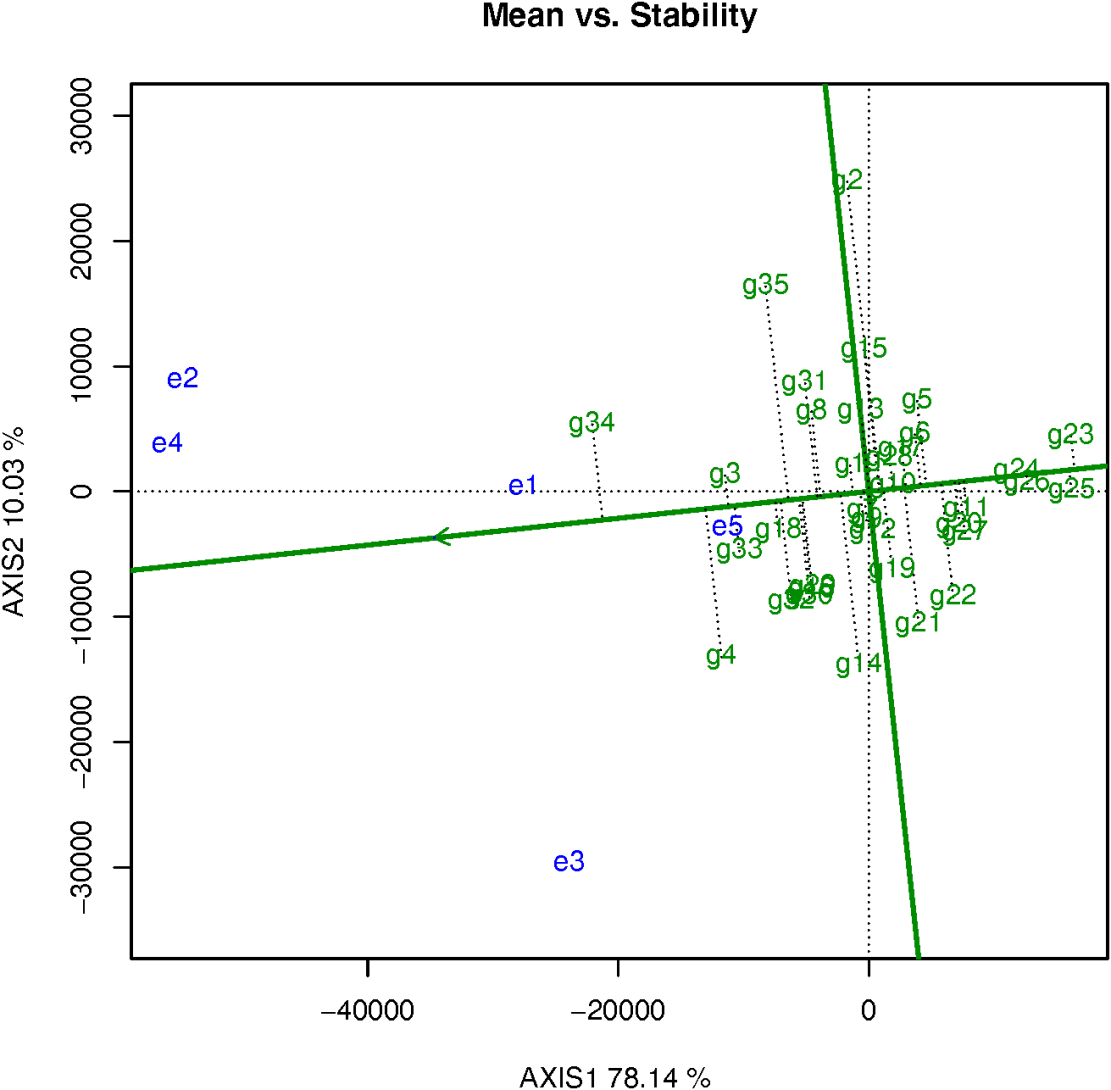
The high and stable yield genotypes.

The angle between the test site and the average environmental axis vector is a measurement of the ability of target environment to be representative. A smaller angle indicates stronger representation. In contrast, a large angle indicates that the representation is weak. The test sites e1 and e5 were ineffective because they could not discriminate genotypes. So, e3 (the angle is bigger than the other four locations) could discriminate but was not representative and could only be used to eliminate unstable genotypes. However, it could not be used to select excellent genotypes (**Figure 6**). Overall, e2 and e4 were most suitable to select genotypes owing to their comprehensive determination of high and stable yields (**Figure 6**).

#### 3.3.3 The high and stable yield genotypes

The line with a single arrow is the average environmental axis. The direction marked by the straight line indicates an increase in production in that direction. The average yield of g34 was the highest, followed by g4, g3, g33 (‘Echo Russet Russet’), and g18 (‘A02267-1Y’) and so on. (**Figure 6**). g34 and g4 were the high-yielding cultivars, but their output varied across environments and was poor in some of them. The average yield of cultivars g1, g7, g9, g10, g12, g13 was close to the total experimental average. A straight line with double arrows perpendicular to the average environmental axis and passing through the origin represents the tendency of each variety to interact with each environment. The length of the short dashed line on the vertical environmental axis represents stable of yield, and the shorter the line, the more stable yield, the longer the line, the more unstable yield. The genotypes g23 (‘A05182-7Y’) had the lowest yields, followed by g25 (‘A06336-2Y’), g26 (‘A07008-4TE’), and g24 (‘A06084-1TE’) (**Figure 6**). Since these genotypes have stable low yields, they could be eliminated from the group of genotypes under study. The genotypes g3 and g33 were identified as the cultivars with the highest and most stable yields.

## 4 Discussion

### 4.1 Development of an evaluation scheme

Currently, there are very few evaluations of comprehensive agronomic traits and qualities of the potatoes used to manufacture French fries. In the past, the genotypes were measured by the experience of researchers and potato breeders for many years and the specific performance of cultivars in the field. There were many non-quantitative factors and subjective factors during the evaluation process. Other studies used a weight coefficients (relative importance, and proportion of various evaluation indicators of a certain tested object) of yield traits as 0.73(Wang, 2017). However, it was 0.301 in this study. This difference is owing to the improvement in yield traits through cultivation techniques in addition to the potential of the variety itself. However, the quality traits of French fries are generally determined by the characteristics of variety. If the French fries do not have a high quality, a high yield is meaningless. Therefore, the weight of yield traits was reduced, and the weight of quality traits was increased to 0.483. AHP is a most widely used technique to assess factors, such as potatoes that are the primary raw materials in a potato chips processing company (Kamble and Raut, 2019), the suitability of land for potato (Iliquín Trigoso et al., 2020), and the soil fertility index suitable for potato production (Bagherzadeh et al., 2018). It consists of estimating the importance of each criterion according to experts’ opinions (Iliquín Trigoso et al., 2020). The AHP technique, which is used to resolve hierarchically structured decision-making problems at different levels, seeks to estimate the relative weight of criteria (Saaty, 1988; Srdjevic, 2005) that could accurately and objectively reflect the quality of materials. Stability and high yield are important indicators to evaluate the genotypes, which are generally measured by the interactions between genotype and environment (Li et al., 2016). These factors play a key role in the selection and evaluation of genotypes that are widely adaptable. Thus, not only the field agronomic traits should be evaluated, but the tuber quality traits should also be evaluated (Liu et al., 2017). It is necessary to use agronomic traits combined with potato processing quality indicators for comprehensive evaluations to identify genotypes that are suitable for processing into French fries in Yunnan. The GGEbiplot has been widely used to evaluate other crops, such as 10 genotypes of winter wheat (Triticum aestivum) in 10 test sites, and comprehensively evaluated the primary genotypic effect G and its interaction effect with the environment GE, which could obtain a reasonable evaluation of the representativeness and discrimination of the test site (Yan et al., 2000).Thus, this study used the two methods AHP and GGEbiplot to evaluate the potato genotypes imported from the US and grown at five sites in Yunnan, China. In addition, the AHP was used to comprehensively evaluate the potato genotypes for their quality, yield, and growth traits, and the GGEbiplot was used to genotype the main effect G and its GE.

### 4.2 The evaluation indicators and the restrictive criteria of the indicators screen

#### 4.2.1 Quality traits

The source of raw potato of different location tubers and genotypes has significant effect on the quality of French fries, this is consistent with the results of Agblor and Scanlonin, 2002. The aim of this study was to screen evaluation indicators and to construct a comprehensive evaluation system of potato genotypes, including the potato cultivars for their suitability for processing into French fries. To achieve commercial success, genotypes for French fries have to fulfill stringent requirements concerning tuber quality traits, such as the color of French fries etc (Bahati-Kajunju, 2021), the length of tuber, the length/width of tuber, appropriate shape, dry matter content, texture changes after frying, and the hardness of French fries after frying (Agblor and Scanlon, 2002; Cunningham et al., 2008). In the past few years, potato genotypes used for French fries were evaluated, but the raw potato tubers all originated from the same site (Abong et al., 2009; Sharara, 2015; Kaur et al., 2021). In these studies, multiple indicators were used, but the indicator was only a simple description, such as a desirable oblong shape, higher dry matter (dry matter of one potato genotype was 21.61%) and a lower content of reducing sugars (reducing sugars content of one potato genotype was 0.06%). However, this method is not suitable to evaluate multiple potato genotypes at numerous sites. The selection of indicators and the critical values are very important for the evaluation system. In one study, the color, texture, taste and smell, general appearance, moisture, fat content, and dark ends as features were used to assess the quality of the French fries processed from 14 potato cultivars (Sawicka et al., 2021). In another study, the crispness, exterior shell, mealiness, moistness, textural variation, and internal appearance were used to assess the quality of the French fries of three processing potato cultivars, including ‘Russet Burbank’, ‘Alpine Russet’, and ‘Clearwater Russet’ (Waxman et al., 2018). And many studies vary in the selection of the critical values of evaluation indicators. For example, tubers for the French fry market are longer, and the best tuber length/width (L/W) ratio of the tubers is 1.75. This is the gold standard for the evaluation of French fries that are processed for McDonald’s in the US, and the L/W ratio should exceed 1.8 (Neilson et al., 2021). The L/W ratio standard for the French fries was 1.5 in a study by Liu et al. (2017). In this study, the critical values of L/W ratio were 1.5, 1.75 and 2.5. If the L/W ratio is small than 1.5, the score was 10. If the L/W ratio< 1.75, the score was 100, and if the L/W ratio > 2.5, the score was 10, A higher overall score was given to genotypes with the desired L/W ratio, based on the L/W ratio is in 1.5~1.0, the tuber is near round and too much flesh is wasted after cutting French fries, and if the L/W ratio > 2.5, French fries is easy to break in the middle. Kaur et al. (2021) noted that the length of tubers > 5 cm was acceptable, and the tuber length that was the most suitable for French fries was 7.9-10.2 cm. In the UK, incremental bonus points are paid on long cultivars when a percentage of tubers > 7.5 cm long (Kirkman, 2007). The width of tubers > 5 cm was acceptable, but penalties will be incurred if the width of tubers is< 5 cm. The dry matter content that is the most suitable for French fries is 19.1-21.6%, and the best dry matter content is ≥ 20% (Kaur et al., 2021). A dry matter content ≤ 19.5% is not acceptable for French fries. Upper limits do not apply, although penalties may be incurred for >25% dry matter during the manufacturing of French fries (Kirkman, 2007). However, others consider that a dry matter content ≥ 20% is acceptable (Abong et al., 2009). A dry matter content > 25% would indicate that a variety could be used to process French fries (Liu et al., 2017). The best dry matter content is 20.7%-24.0%, which is the gold standard for the evaluation of processing McDonald’s fries in the US. In this study, we found if the value of dry matter content< 19.5%, the French fries color of this cultivars was dark, and if the value of dry matter content >25%, the French fries color of this cultivars was slight dark, and the internal appearance of this cultivars was additional hollowing. So when the value was< 19.5%; the score was 10. A value of 25% resulted in a score of 100, and a value > 25% resulted in a decreased score. In conclusion, the L/W ratio is near 1.75, dry matter content near 25% and the tuber length is more than 5cm is more suitable for French fries.

#### 4.2.2 Yield traits

Yield traits offer important information about potato cultivars, such as the higher the ratio of large tubers, the higher the yield. Yield components are also important factors for growers to obtain monetary returns. In this study, the highest value of the ratio of large tubers and yield in each location resulted in a score of 100, and the yield and the ratio of large tuber data of other genotypes were standardized. In addition, the uniformity of tubers is very important for processing French fries to ensure the uniformity of the manufacturing process. One UK manufacturer established the indicators, such as tuber count per 10 kg, and the acceptable tuber count range is 34-67 per 10 kg – an indicator of the distribution of tuber sizes (Kirkman, 2007), which corresponds to the weight per marketable tuber of 294-149 g per tuber. In this study, the weight per marketable tuber was used as one evaluating indicator. Since the ratio of large tubers determines the market price, the tuber consistency and small tuber ratio are the restrictive standards (minimum or maximum standards) for the French fry companies to purchase raw potato tubers. If the proportion of tubers< 5 cm wide exceeds 12%, the lots could be rejected (Kirkman, 2007). In this study, a small tuber ratio was used as a limiting standard. If the small tuber ratio exceeded 12%, the score was 10 out of a total of 100. In summary, the ratio of large tubers, tuber consistency and a small tuber ratio was more important or had a higher priority than the yield factor in our study, but in the past, the small tuber ratio was not selected as one indicator. So the selected of yield traits indicators and their limiting standard were more reasonable.

#### 4.2.3 Agronomic traits

The important agronomic traits include a wide adaptability for characteristics, such as maturity, plant vigor and late blight resistance (AUDPC) (Jansky et al., 2009; Fajardo et al., 2013; Jansky et al., 2013), that contribute to the overall performance of cultivars. In addition, many cultivars differ in plant maturity, vigor and resistance to late blight (AUDPC) under different environments (Johansen et al., 1967). Collins et al., 1999 reported that the plant maturity and vigor strongly correlate with late blight resistance. In our study, late blight is the first major disease to affect the spring potatoes in Yunnan Province, China. Plants that have stronger resistance to late blight, mature later and are more vigorous. However, the potato genotypes imported from the US were less resistance to late blight comparing with the two control cultivars. In addition, the potato genotypes imported from the US are sensitive to day length and not as vigorous as the cultivars adapted to day length of Yunnan Province, China. Thus, the best plant vigor value was 9, their score was 100, and the best plant maturity value was 1. The resistance to late blight of potato was calculated by AUDPC, and a lower AUDPC indicates a stronger resistance to late blight (Forbes et al., 2014). Furthermore, the late blight of all the potato genotypes in this study was chemically controlled, and the incidence of late blight was investigated after chemical control. However, since late blight was not effectively controlled by chemicals in XW, the disease developed rapidly, and the seedlings rapidly died, resulting in low yields.

In our study, the results of AHP and GGEbiplot are highly consistent. The potato genotypes with high comprehensive scores in the AHP are highly adaptable, stable, and high yielding in the GGEbiplot, which is consistent with the performance of the potato genotypes in the field. In addition, the quality of the French Fries is also excellent, such as the g3 ranked top, g23 and g26 ranked bottom by using AHP in 5 sites, and g3 also was showed higher and stable yields, g23 and g26 were also showed lower and unstable yield by using the GGEbiplot. This showed that the results of a comprehensive analysis of genotypes using AHP and GGEbiplot analyses are reasonable and effective, and the established potato genotypes evaluation system could be used as the basis for future evaluation and the identification of cultivars.

## 5 Conclusions

Eleven indicators for quality traits, yield traits and agronomic traits were selected as the specific evaluation indicators in this study. The weights coefficients (relative importance, and proportion of various evaluation indicators of a certain tested object) of quality traits, yield traits and agronomic traits were 0.483, 0.301 and 0.216, respectively, and discriminated significantly from the schemes applied so far. The 35 potato genotypes were evaluated by using AHP and GGEbiplot analysis in Yunnan, China. The cultivars with high and stable yield, good quality of French fries, suitable tuber length/width ratio, and longer tubers were selected for more suitable growing and high quality of French fries in Yunnan. However, as no results exceed the standard cultivar g3, further cultivars will be evaluated to test their suitable for planting and French fries in Yunnan. Furthermore, reasonable suggestions for the exclusion of test sites without affecting the evaluation of genotypes were provided and help to save costs.

## Data availability statement

The raw data supporting the conclusions of this article will be made available by the authors, without undue reservation.

## Author contributions

SL: Investigation, Data collation and analysis, Writing – Original Draft. WJ: Investigation the field data. YY: Improvement of French fries frying method and evaluation method of French fries quality. LL: Tissue cultured plant expanded. JW and RN: Provided the *In vitro* pathogen-free plantlets and revised the manuscript. LB: The Pre-Elite potato seed produced and the experiment in e5 was completed. ZY: The experiment in e2 was completed. ZL: The experiment in e3 was completed. PH: The experiment in e4 was completed. YX: The experiment in e5 was completed. XL: Project administration. All authors contributed to the article and approved the submitted version.

## Supplementary material

Supplementary Figure 1 The disribution of experimental sites in Yunnan Province, China. e1: Huize (HZ), e2: Luquan (LQ), e3-1: Zhaotong (ZT), e3-2: Zhaotong (ZT), e4: Lijiang (LJ), e5: Xuanwei (XW);The same as bellow.

Supplementary Figure 2 Process flow chart of the French fries.

Supplementary Figure 3 The tubers length/width of the different genotypes at e1, e2, e3-1, e3-2 in 2019 and 2020.
