# Supplementary figures and images for: An evaluation based on the analytic hierarchy process and GGEbiplot on French fry potato genotypes in Yunnan, China
Source: Front Plant Sci. 2023 Sep 18;14:1159848. doi: 10.3389/fpls.2023.1159848 (PMC10544891; doi:10.3389/fpls.2023.1159848)

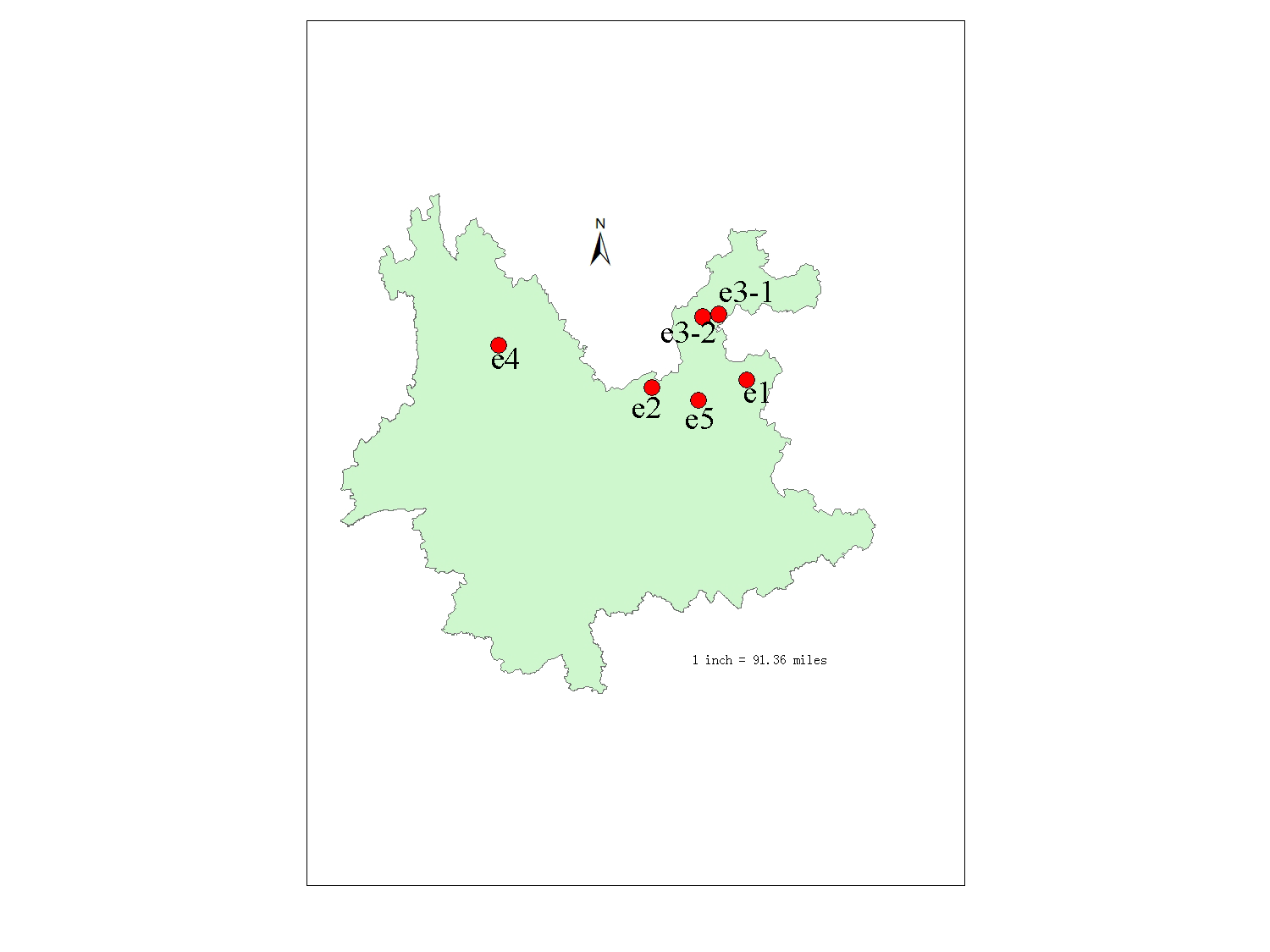

Supplement: Supplementary Figure 1 — The disribution of experimental sites in Yunnan Province, China. e1: Huize (HZ), e2: Luquan (LQ), e3-1: Zhaotong (ZT), e3-2: Zhaotong (ZT), e4: Lijiang (LJ), e5: Xuanwei (XW);The same as bellow. [file Image_1.tiff]

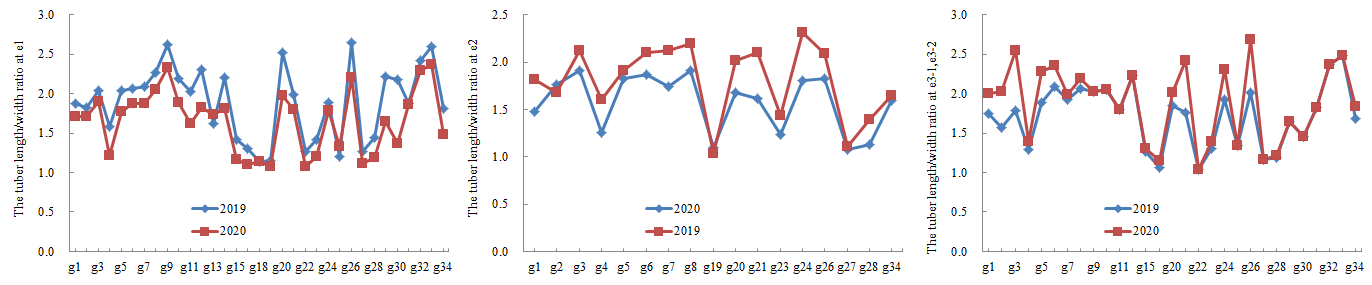

Supplement: Supplementary Figure 3 — The tubers length/width of the different genotypes at e1, e2, e3-1, e3-2 in 2019 and 2020. [file Image_3.tif]
