# Supplementary material for: An evaluation based on the analytic hierarchy process and GGEbiplot on French fry potato genotypes in Yunnan, China
Source: Front Plant Sci. 2023 Sep 18;14:1159848. doi: 10.3389/fpls.2023.1159848 (PMC10544891; doi:10.3389/fpls.2023.1159848)
Supplement: Supplementary file 4 [file DataSheet_1.docx]

Supplementary Table 1: The name/ breeding code of the experimental genotypes

| **The serial number** | **Name / Breeding code** | **The serial number** | **Name / Breeding code** | |
| --- | --- | --- | --- | --- |
| **g1** | Premier Russet（A93157-6LS） | **g19** | A02267-5PY | |
| **g2** | Palisade Russet（A97066-42LB） | **g20** | A03141-6 | |
| **g3** | Defender（A90586-11） | **g21** | A03921-2 | |
| **g4** | Yukon Gem（NDA5507-3Y） | **g22** | A05180-3PY | |
| **g5** | GemStar Russet（A9014-2） | **g23** | A05182-7Y | |
| **g6** | Targhee Russet（A01010-1） | **g24** | A06084-1TE | |
| **g7** | Alpine Russet（A9305-10） | **g25** | A06336-2Y | |
| **g8** | Mountain Gem Russet（A03158-2TE） | **g26** | A07008-4TE | |
| **g9** | Sage Russet（AO96164-1） | **g27** | COA07365-4RY | |
| **g10** | Castle Russet（POR06V12-3） | **g28** | NDA081451CB-1CY | |
| **g11** | Blazer Russet（A8893-1） | **g29** | PA99N2-1 | |
| **g12** | Owyhee Russet（AO96160-3） | **g30** | PA99N82-4 | |
| **g13** | Alturas（A82360-7） | **g31** | AO03123-2 | |
| **g14** | Highland Russet（A9045-7） | **g32** | AO96305-3 | |
| **g15** | Smilin’ Eyes（POR02PG26-5） | **g33** | Echo Russet（AO96141-3） | |
| **g16** | Huckleberry Gold（A99326-1PY） | **g34** | YS401 | |
| **g17** | A00188-3C | **g35** | Cooperation 88 | |
| **g18** | A02267-1Y |  |  |  |

Supplementary Table 2: The investigation standard of the vigor of potato plants

| **Scale** | **State** | **Description** |
| --- | --- | --- |
| **1** | Very weak | All the plants are small (<20 cm), few leaves, weak plants, very thin stems and/or light green color. |
| **3** | Weak | 75% of the plants are small (<20 cm) or all the plants are between 20-30cm, the plants have few leaves, thin stems and /or light green color. |
| **5** | Medium | Intermediate or normal. |
| **7** | Vigorous | 75% of the plants are over 50cm, robust with foliage of dark green color, thick stems and leaves very well developed. |
| **9** | Very vigorous | All the plants are over 70cm and ground coverage is complete. The plants  are robust, with thick stems and abundant foliage of dark green color. |

Supplementary Table 3: The standard to evaluate the maturation of potato plants

| **Scale** | **1** | **3** | **5** | **7** | **9** |
| --- | --- | --- | --- | --- | --- |
| **Description** | plants green | 25% foliage yellow | 50% foliage yellow | 75% foliage yellow | 100% foliage burned |

Supplementary Table 4: The proportion criteria for pair-wise comparisons

| Definition | Equal importance | Moderate importance | Significantly more important | Very  Strong importance | Extreme importance |
| --- | --- | --- | --- | --- | --- |
| Proportion criteria | 1 | 1.2 | 1.4 | 1.6 | 1.8 |

Supplementary Table 5: Average random index (RI) based on size (Kumar et al., 2009)

| n | 1 | 2 | 3 | 4 | 5 | 6 | 7 | 8 | 9 | 10 |
| --- | --- | --- | --- | --- | --- | --- | --- | --- | --- | --- |
| RI | 0 | 0 | 0.52 | 0.89 | 1.11 | 1.25 | 1.35 | 1.40 | 1.45 | 1.49 |

Supplementary Table 6: Sensory evaluation criteria (Waxman et al., 2019)

| **Internal Appearance** | | **Crispness** | |
| --- | --- | --- | --- |
| **1** | severely raw | 1 | no crispness |
| **2** | slightly raw | 2 | nearly no crispness |
| **3** | partially cooked | 3 | lacking crispness |
| **4** | less than fluffy | 4 | less crispness than 5 |
| **5** | fluffy cooked appearance | 5 | light, but notable pleasing crispness |
| **6** | slight pulling from edges | 6 | light crunch |
| **7** | additional hollowing | 7 | distinctive crunch |
| **8** | serious hollowing | 8 | undesirable crunch |
| **9** | most severe hollowing | 9 | severely undesirable crunch |
| **Exterior Shell** | | Mealiness | |
| **1** | no exertion | 1 | no granules |
| **2** | slight exertion | 2 | very small granules |
| **3** | less exertion | 3 | small granules |
| **4** | exertion less than desired | 4 | smaller but acceptable granules |
| **5** | slight tug to pull from teeth | 5 | small slightly firm granules |
| **6** | slightly more exertion | 6 | slightly larger granules |
| **7** | more exertion | 7 | large, coarse granules |
| **8** | much more exertion | 8 | larger, coarser granules |
| **9** | too tough | 9 | largest, coarsest granules |
| **Moistness** | | Textural Variation | |
| **1** | extremely dry | 1 | Not applicable. Scoring begins at 5 |
| **2** | very dry | 2 | Not applicable. Scoring begins at 5 |
| **3** | dry | 3 | Not applicable. Scoring begins at 5 |
| **4** | slightly drier than desired | 4 | Not applicable. Scoring begins at 5 |
| **5** | slightly dry, hint of moisture | 5 | uniform color, acceptable length |
| **6** | moister but acceptable | 6 | Slight variation in color and shorter fries |
| **7** | additional moisture | 7 | More variation in color and shorter fries |
| **8** | much more moisture | 8 | serious dark color and shorts |
| **9** | extreme moisture | 9 | extreme dark color and shorts |

Supplementary Table 7: Results of non-dimensional and comprehensive scores of different traits for the imported potato genotypes in e1.

| **e1** | **The score of 11 components** | | | | | | | | | | | **Total score** |
| --- | --- | --- | --- | --- | --- | --- | --- | --- | --- | --- | --- | --- |
|  | **Quality of french fries** | **Length /width of tubers** | **Dry matter content（%）** | **Length of tubers（cm）** | **The ratio of big tuber** | **The weight per marketable tuber**  **（g / one tuber）** | **Small tuber ratio（width<50mm）** | **Yield（Kg/**  **hm^2^）** | **Maturity** | **Plant vigor** | **Late blight resistance（AUDPC）** |  |
| **g31** | 11.79 | 10.54 | 9.80 | 7.48 | 9.06 | 7.29 | 4.02 | 3.67 | 2.95 | 7.37 | 1.80 | 75.78 |
| **g34** | 12.26 | 7.88 | 9.82 | 8.26 | 8.76 | 7.68 | 0.68 | 4.32 | 6.07 | 4.61 | 5.27 | 75.60 |
| **g3** | 10.03 | 9.13 | 10.11 | 8.52 | 10.72 | 7.68 | 3.94 | 5.33 | 4.59 | 4.06 | 1.26 | 75.36 |
| **g2** | 11.61 | 12.10 | 9.79 | 7.11 | 9.30 | 7.68 | 1.15 | 4.04 | 6.56 | 4.61 | 1.34 | 75.28 |
| **g29** | 11.70 | 9.70 | 10.48 | 7.79 | 8.60 | 7.68 | 2.07 | 4.05 | 4.26 | 7.37 | 0.93 | 74.64 |
| **g1** | 12.72 | 11.65 | 10.05 | 7.05 | 8.83 | 7.29 | 2.39 | 3.60 | 0.98 | 4.06 | 1.56 | 70.19 |
| **g35** | 12.35 | 6.14 | 9.96 | 7.08 | 7.62 | 7.05 | 0.35 | 3.80 | 7.55 | 2.40 | 4.46 | 68.76 |
| **g7** | 10.49 | 8.90 | 9.69 | 7.55 | 7.69 | 7.68 | 1.30 | 3.33 | 3.61 | 6.82 | 1.50 | 68.57 |
| **g30** | 12.17 | 12.01 | 9.78 | 7.06 | 5.15 | 5.48 | 1.36 | 3.75 | 0.66 | 6.27 | 1.32 | 65.01 |
| **g20** | 12.54 | 4.99 | 11.11 | 7.64 | 8.28 | 6.89 | 3.80 | 2.70 | 1.64 | 4.06 | 1.20 | 64.84 |
| **g21** | 12.17 | 10.20 | 1.23 | 7.08 | 9.51 | 7.68 | 3.37 | 3.11 | 3.94 | 5.16 | 0.96 | 64.41 |
| **g32** | 9.94 | 3.37 | 10.76 | 8.53 | 8.11 | 7.05 | 3.21 | 3.80 | 1.64 | 6.27 | 1.37 | 64.03 |
| **g14** | 10.59 | 8.48 | 1.23 | 7.91 | 9.60 | 7.68 | 4.62 | 3.76 | 1.97 | 6.27 | 1.17 | 63.27 |
| **g13** | 12.26 | 8.98 | 1.23 | 7.38 | 8.75 | 7.68 | 0.82 | 3.78 | 5.25 | 5.72 | 0.99 | 62.84 |
| **g8** | 11.14 | 6.25 | 1.23 | 7.81 | 9.76 | 7.68 | 3.37 | 4.02 | 3.28 | 6.82 | 1.43 | 62.79 |
| **g11** | 11.89 | 11.29 | 10.45 | 6.99 | 6.54 | 6.88 | 0.68 | 2.74 | 0.66 | 4.06 | 0.53 | 62.69 |
| **g10** | 10.03 | 8.10 | 11.45 | 7.67 | 6.21 | 6.79 | 0.35 | 3.11 | 1.97 | 5.16 | 1.58 | 62.42 |
| **g6** | 10.31 | 9.13 | 9.79 | 7.39 | 5.83 | 5.95 | 0.33 | 2.97 | 2.30 | 6.27 | 1.85 | 62.11 |
| **g5** | 12.72 | 10.07 | 1.23 | 6.92 | 7.53 | 7.40 | 0.81 | 3.43 | 3.28 | 6.82 | 1.21 | 61.43 |
| **g33** | 11.89 | 1.49 | 10.50 | 8.23 | 5.88 | 5.77 | 0.40 | 3.79 | 2.62 | 7.37 | 2.04 | 59.99 |
| **g24** | 10.96 | 11.14 | 9.82 | 5.95 | 6.01 | 5.46 | 1.52 | 2.42 | 0.98 | 4.06 | 0.45 | 58.78 |
| **g9** | 11.52 | 1.61 | 11.21 | 8.81 | 8.43 | 6.66 | 0.64 | 3.43 | 0.33 | 4.61 | 1.26 | 58.51 |
| **g4** | 10.40 | 1.23 | 10.08 | 6.42 | 6.38 | 6.54 | 2.48 | 4.13 | 0.98 | 6.82 | 0.59 | 56.07 |
| **g16** | 9.84 | 1.23 | 10.76 | 5.79 | 6.55 | 7.16 | 1.38 | 3.87 | 0.98 | 6.27 | 1.30 | 55.15 |
| **g12** | 10.77 | 7.67 | 1.23 | 7.57 | 7.42 | 6.54 | 0.64 | 2.89 | 1.97 | 5.72 | 0.86 | 53.28 |
| **g22** | 8.17 | 1.23 | 11.82 | 4.75 | 6.05 | 6.89 | 1.26 | 2.92 | 1.31 | 5.72 | 1.00 | 51.12 |
| **g26** | 11.52 | 2.40 | 10.29 | 7.63 | 5.13 | 5.68 | 0.38 | 1.75 | 1.31 | 3.50 | 1.35 | 50.95 |
| **g19** | 10.96 | 1.23 | 11.67 | 5.14 | 5.47 | 5.22 | 0.64 | 2.88 | 0.98 | 4.61 | 1.30 | 50.10 |
| **g18** | 10.96 | 1.23 | 1.23 | 5.68 | 6.95 | 6.67 | 3.26 | 4.03 | 2.30 | 6.27 | 1.49 | 50.08 |
| **g28** | 7.62 | 1.23 | 10.33 | 5.43 | 5.62 | 5.80 | 0.97 | 3.03 | 3.28 | 5.72 | 0.83 | 49.85 |
| **g17** | - | 1.23 | 10.24 | 5.14 | 8.14 | 6.59 | 2.89 | 2.72 | 2.62 | 5.16 | 1.47 | 46.21 |
| **g15** | 8.17 | 1.23 | 9.82 | 5.34 | 4.89 | 4.70 | 0.42 | 3.18 | 0.33 | 4.61 | 1.00 | 43.69 |
| **g23** | 9.84 | 1.23 | 10.35 | 5.15 | 1.87 | 3.62 | 0.18 | 1.34 | 0.66 | 2.40 | 1.28 | 37.90 |
| **g27** | 9.29 | 1.23 | 1.23 | 5.07 | 6.33 | 5.31 | 0.36 | 2.31 | 0.98 | 3.50 | 1.77 | 37.39 |
| **g25** | 8.17 | 1.23 | 1.23 | 4.38 | 0.66 | 3.74 | 0.00 | 1.27 | 0.98 | 2.40 | 0.94 | 25.02 |

Supplementary Table 8: Results of non-dimensional and comprehensive scores of the different traits for imported potato genotypes in e2.

| e2 | **The score of 11 components** | | | | | | | | | | | **Total score** |
| --- | --- | --- | --- | --- | --- | --- | --- | --- | --- | --- | --- | --- |
|  | **Quality of french fries** | **Length /width of tubers** | **Dry matter content（%）** | **Length of tubers（cm）** | **The ratio of big tuber** | **The weight per marketable tuber**  **（g / one tuber）** | **Small tuber ratio（width<50mm）** | **Yield（Kg/**  **hm^2^）** | **Maturity** | **Plant vigor** | **Late blight resistance（AUDPC）** |  |
| **g1** | 13.74 | 7.79 | 10.77 | 6.99 | 9.76 | 7.68 | 3.22 | 3.00 | - | 7.37 | 1.21 | 71.53 |
| **g7** | 12.63 | 9.68 | 11.00 | 7.64 | 9.63 | 7.68 | 2.91 | 2.72 | - | 6.27 | 1.09 | 71.24 |
| **g32** | 13.74 | 8.00 | 10.04 | 7.35 | 8.74 | 7.68 | 3.43 | 3.34 | - | 7.37 | 1.02 | 70.72 |
| **g3** | 10.91 | 8.38 | 10.63 | 8.81 | 10.72 | 7.68 | 5.05 | 3.82 | - | 3.50 | 1.18 | 70.67 |
| **g21** | 12.07 | 10.72 | 11.90 | 6.54 | 7.76 | 7.68 | 3.05 | 2.07 | - | 6.27 | 1.21 | 69.27 |
| **g10** | 13.00 | 11.72 | 10.20 | 7.12 | 7.13 | 7.68 | 0.96 | 2.32 | - | 7.37 | 1.16 | 68.65 |
| **g5** | 12.26 | 10.57 | 10.17 | 7.00 | 9.24 | 7.68 | 2.16 | 2.57 | - | 5.72 | 1.00 | 68.36 |
| **g34** | 11.13 | 6.61 | 10.12 | 6.84 | 9.78 | 7.68 | 3.02 | 5.33 | - | 1.98 | 4.78 | 67.27 |
| **g6** | 12.91 | 8.83 | 9.92 | 7.45 | 7.58 | 7.68 | 0.54 | 2.62 | - | 7.37 | 1.19 | 66.09 |
| **g2** | 12.26 | 11.09 | 10.97 | 7.23 | 7.99 | 7.68 | 0.64 | 2.83 | - | 3.50 | 0.97 | 65.16 |
| **g33** | 13.56 | 4.09 | 9.95 | 7.87 | 8.23 | 7.68 | 3.17 | 3.08 | - | 6.27 | 1.04 | 64.94 |
| **g31** | 13.56 | 9.78 | 1.23 | 6.95 | 8.69 | 7.68 | 3.99 | 3.04 | - | 7.37 | 1.18 | 63.47 |
| **g20** | 11.61 | 10.89 | 11.57 | 6.61 | 6.07 | 6.98 | 0.64 | 1.58 | - | 5.16 | 1.18 | 62.28 |
| **g14** | 13.37 | 8.66 | 1.23 | 6.84 | 10.00 | 7.68 | 5.05 | 2.12 | - | 6.27 | 0.88 | 62.10 |
| **g26** | 12.40 | 9.23 | 10.00 | 6.61 | 5.94 | 6.40 | 3.96 | 1.09 | - | 5.16 | 1.02 | 61.80 |
| **g8** | 12.91 | 7.82 | 1.23 | 7.47 | 9.36 | 7.68 | 2.81 | 3.06 | - | 6.82 | 1.00 | 60.17 |
| **g13** | 12.07 | 12.31 | 1.23 | 6.75 | 9.33 | 7.68 | 2.50 | 2.59 | - | 4.61 | 1.11 | 60.17 |
| **g4** | 10.82 | 1.23 | 10.47 | 6.27 | 6.56 | 7.57 | 4.41 | 3.07 | - | 6.82 | 1.07 | 58.29 |
| **g29** | 12.26 | 1.23 | 10.87 | 5.49 | 7.05 | 7.68 | 2.04 | 2.81 | - | 7.37 | 1.18 | 57.98 |
| **g12** | 12.82 | 11.02 | 1.23 | 6.81 | 7.90 | 7.68 | 0.11 | 2.09 | - | 6.27 | 1.16 | 57.09 |
| **g9** | 12.63 | 7.46 | 1.23 | 7.71 | 8.88 | 7.68 | 1.17 | 2.43 | - | 5.72 | 0.97 | 55.87 |
| **g18** | 14.39 | 1.23 | 1.23 | 4.91 | 9.29 | 7.68 | 5.64 | 2.83 | - | 7.37 | 0.97 | 55.55 |
| **g24** | 12.40 | 7.83 | 10.65 | 6.59 | 4.18 | 5.24 | 0.15 | 1.12 | - | 4.06 | 1.16 | 53.36 |
| **g28** | 12.63 | 1.23 | 9.98 | 4.99 | 4.10 | 5.98 | 0.30 | 2.60 | - | 5.72 | 1.00 | 48.54 |
| **g30** | 13.37 | 1.23 | 1.23 | 5.80 | 6.32 | 7.68 | 0.30 | 2.92 | - | 7.37 | 1.11 | 47.34 |
| **g11** | 12.07 | 5.36 | 1.23 | 6.40 | 5.58 | 7.25 | 0.99 | 1.42 | - | 5.16 | 0.95 | 46.43 |
| **g17** | - | 1.23 | 9.72 | 5.11 | 6.72 | 7.68 | 1.97 | 2.59 | - | 6.27 | 1.14 | 42.43 |
| **g16** | - | 1.23 | 9.65 | 4.73 | 6.83 | 6.61 | 4.10 | 2.21 | - | 5.72 | 0.53 | 41.61 |
| **g35** | 12.44 | 1.23 | 1.23 | 6.32 | 5.69 | 7.03 | 0.25 | 2.83 | - | 0.74 | 3.77 | 41.53 |
| **g15** | 13.74 | 1.23 | 1.23 | 5.00 | 4.54 | 6.16 | 0.29 | 2.40 | - | 5.72 | 0.84 | 41.17 |
| **g22** | - | 1.23 | 10.12 | 4.12 | 7.22 | 6.56 | 4.70 | 1.33 | - | 5.16 | 0.70 | 41.16 |
| **g19** | - | 1.23 | 11.03 | 4.29 | 5.29 | 6.44 | 2.79 | 2.02 | - | 5.72 | 0.88 | 39.69 |
| **g23** | 9.29 | 1.23 | 0.15 | 4.87 | 1.12 | 4.02 | 0.00 | 0.78 | - | 4.06 | 0.44 | 25.95 |
| **g25** | - | 1.23 | 9.96 | 4.40 | 0.94 | 4.72 | 0.15 | 0.70 | - | 2.95 | 0.70 | 25.76 |
| **g27** | - | 1.23 | 0.15 | 4.23 | 4.61 | 5.03 | 1.76 | 1.46 | - | 5.16 | 0.84 | 24.48 |

Note: The values of maturity data of e2 is missing, and “-” of the quality of French fries means the tuber is round or (length to width ratio close to 1), the same as bellow.

Supplementary Table 9: Results of the non-dimensional and comprehensive scores of different traits for imported potato genotypes in e3

| **e3** | **The score of 11 components** | | | | | | | | | | | **Total score** |
| --- | --- | --- | --- | --- | --- | --- | --- | --- | --- | --- | --- | --- |
|  | **Quality of french fries** | **Length /width of tubers** | **Dry matter content（%）** | **Length of tubers（cm）** | **The ratio of big tuber** | **The weight per marketable tuber**  **（g / one tuber）** | **Small tuber ratio（width<50mm）** | **Yield（Kg/**  **hm^2^）** | **Maturity** | **Plant vigor** | **Late blight resistance（AUDPC）** |  |
| **g1** | 12.84 | 10.47 | 10.06 | 7.22 | 10.04 | 7.68 | 0.97 | 3.71 | 3.28 | 5.16 | 3.86 | 75.28 |
| **g2** | 11.59 | 11.55 | 9.87 | 6.81 | 8.97 | 7.68 | 0.26 | 1.99 | 4.59 | 5.72 | 4.64 | 73.66 |
| **g21** | 11.14 | 7.34 | 10.36 | 7.96 | 10.30 | 7.68 | 2.20 | 4.31 | 3.28 | 3.50 | 4.21 | 72.28 |
| **g11** | 10.77 | 11.56 | 9.85 | 7.10 | 10.06 | 7.68 | 1.10 | 3.30 | 2.62 | 5.16 | 2.98 | 72.19 |
| **g34** | 10.33 | 12.16 | 1.23 | 6.96 | 9.28 | 7.68 | 0.31 | 4.73 | 8.86 | 3.78 | 5.27 | 70.58 |
| **g3** | 10.82 | 6.11 | 10.11 | 8.81 | 9.82 | 7.68 | 0.40 | 4.30 | 4.26 | 2.95 | 5.27 | 70.54 |
| **g10** | 11.52 | 7.79 | 10.40 | 6.96 | 8.72 | 7.68 | 0.21 | 3.57 | 3.77 | 4.61 | 4.99 | 70.23 |
| **g20** | 11.61 | 9.65 | 10.45 | 6.67 | 9.13 | 7.68 | 0.28 | 3.46 | 2.87 | 4.61 | 3.64 | 70.05 |
| **g5** | 10.68 | 7.41 | 9.57 | 7.68 | 9.57 | 7.68 | 0.36 | 3.08 | 3.20 | 6.27 | 4.43 | 69.92 |
| **g8** | 9.29 | 6.71 | 10.10 | 6.80 | 9.52 | 7.68 | 0.35 | 3.56 | 3.69 | 7.37 | 4.73 | 69.78 |
| **g32** | 12.07 | 3.24 | 11.17 | 7.62 | 9.80 | 7.68 | 0.40 | 4.78 | 3.28 | 4.61 | 4.71 | 69.37 |
| **g14** | 10.68 | 11.53 | 1.23 | 7.63 | 10.72 | 7.68 | 4.21 | 4.72 | 3.20 | 3.50 | 3.91 | 69.00 |
| **g26** | 10.08 | 3.48 | 10.24 | 7.31 | 10.18 | 7.68 | 1.67 | 2.94 | 3.53 | 6.27 | 4.93 | 68.30 |
| **g12** | 11.70 | 5.24 | 9.63 | 7.97 | 10.08 | 7.68 | 1.20 | 3.92 | 3.20 | 2.95 | 4.39 | 67.94 |
| **g31** | 10.87 | 11.32 | 9.64 | 6.56 | 9.19 | 7.68 | 0.29 | 3.42 | 2.71 | 3.50 | 2.73 | 67.91 |
| **g7** | 10.96 | 9.22 | 1.23 | 7.44 | 9.84 | 7.68 | 0.64 | 3.96 | 5.50 | 5.72 | 5.27 | 67.44 |
| **g33** | 12.35 | 1.58 | 10.39 | 8.57 | 10.10 | 7.68 | 1.28 | 4.64 | 2.95 | 2.95 | 4.30 | 66.79 |
| **g29** | 12.44 | 7.69 | 9.65 | 6.74 | 9.83 | 7.68 | 0.64 | 4.56 | 2.13 | 3.50 | 1.82 | 66.69 |
| **g30** | 13.74 | 1.23 | 9.64 | 6.20 | 10.22 | 7.68 | 1.87 | 4.67 | 2.79 | 4.61 | 3.93 | 66.58 |
| **g13** | 12.91 | 11.59 | 1.23 | 6.73 | 9.76 | 7.68 | 0.39 | 3.33 | 3.28 | 5.16 | 4.15 | 66.21 |
| **g25** | 11.33 | 1.23 | 9.67 | 5.59 | 9.22 | 7.59 | 0.30 | 2.76 | 2.95 | 7.37 | 4.25 | 62.26 |
| **g6** | 12.44 | 5.27 | 1.23 | 7.17 | 9.51 | 7.23 | 0.35 | 3.27 | 4.10 | 3.50 | 4.99 | 59.06 |
| **g9** | 7.06 | 8.21 | 1.23 | 6.73 | 9.85 | 7.68 | 0.64 | 3.91 | 2.87 | 5.72 | 3.24 | 57.12 |
| **g35** | 11.19 | 1.23 | 1.23 | 5.61 | 8.56 | 7.48 | 0.19 | 2.85 | 7.83 | 2.26 | 5.27 | 53.71 |
| **g24** | 11.19 | 6.89 | 1.23 | 7.01 | 8.79 | 7.23 | 0.22 | 2.86 | 2.13 | 3.50 | 1.73 | 52.79 |
| **g4** | 10.22 | 1.23 | 1.23 | 5.86 | 10.21 | 7.68 | 1.82 | 5.33 | 2.71 | 2.40 | 3.65 | 52.34 |
| **g19** | 9.29 | 1.23 | 1.23 | 5.31 | 10.13 | 6.60 | 1.40 | 4.07 | 3.69 | 3.50 | 5.06 | 51.52 |
| **g27** | 9.84 | 1.23 | 1.23 | 4.67 | 7.94 | 6.55 | 0.08 | 3.52 | 3.53 | 6.27 | 4.86 | 49.73 |
| **g17** | - | 1.23 | 10.37 | 4.67 | 8.49 | 7.68 | 0.17 | 3.42 | 3.28 | 5.72 | 4.42 | 49.46 |
| **g23** | 7.62 | 1.23 | 1.23 | 4.91 | 8.61 | 6.48 | 0.19 | 2.43 | 2.54 | 6.82 | 2.98 | 45.05 |
| **g18** | - | 1.23 | 1.23 | 5.01 | 9.93 | 7.68 | 0.64 | 4.33 | 3.69 | 5.72 | 5.06 | 44.51 |
| **g16** | - | 1.23 | 1.23 | 5.61 | 10.29 | 7.68 | 2.20 | 4.43 | 3.28 | 2.95 | 4.50 | 43.42 |
| **g22** | - | 1.23 | 1.23 | 5.02 | 9.45 | 7.68 | 0.34 | 3.89 | 3.28 | 6.27 | 4.59 | 42.98 |
| **g15** | 9.29 | 1.23 | 1.23 | 5.26 | 8.26 | 6.02 | 0.13 | 2.84 | 1.80 | 5.16 | 0.53 | 41.76 |
| **g28** | 7.06 | 1.23 | 1.23 | 4.50 | 7.49 | 5.66 | 0.00 | 3.50 | 2.46 | 5.72 | 2.55 | 41.41 |

Supplementary Table 10: Results of non-dimensional and comprehensive scores of different traits for the imported potato genotypes in e4.

| e4 | **The score of 11 components** | | | | | | | | | | | **Total score** |
| --- | --- | --- | --- | --- | --- | --- | --- | --- | --- | --- | --- | --- |
|  | **Quality of french fries** | **Length /width of tubers** | **Dry matter content（%）** | **Length of tubers（cm）** | **The ratio of big tuber** | **The weight per marketable tuber**  **（g / one tuber）** | **Small tuber ratio（width<50mm）** | **Yield（Kg/**  **hm^2^）** | **Maturity** | **Plant vigor** | **Late blight resistance（AUDPC）** |  |
| **g34** | 12.12 | 11.57 | 10.47 | 7.26 | 9.82 | 7.68 | 2.90 | 5.33 | 6.89 | 4.33 | 5.04 | 83.41 |
| **g3** | 13.37 | 10.01 | 11.18 | 8.81 | 10.72 | 7.68 | 3.63 | 3.66 | 5.91 | 4.61 | 3.57 | 83.12 |
| **g2** | 12.82 | 7.95 | 9.54 | 6.17 | 9.69 | 7.68 | 4.44 | 3.27 | 5.91 | 5.16 | 3.35 | 75.98 |
| **g7** | 13.00 | 8.47 | 10.75 | 6.27 | 10.20 | 7.68 | 3.84 | 2.40 | 3.94 | 6.82 | 2.27 | 75.64 |
| **g8** | 12.07 | 10.14 | 10.30 | 7.38 | 8.90 | 7.68 | 1.43 | 3.23 | 3.94 | 6.27 | 2.92 | 74.26 |
| **g5** | 12.82 | 12.04 | 10.43 | 6.61 | 9.36 | 7.68 | 1.84 | 1.51 | 3.94 | 5.16 | 1.75 | 73.14 |
| **g9** | 11.89 | 7.80 | 10.03 | 7.73 | 8.15 | 7.68 | 2.85 | 2.59 | 3.94 | 7.37 | 2.98 | 73.00 |
| **g10** | 14.86 | 10.24 | 10.74 | 6.79 | 6.13 | 7.67 | 0.64 | 2.26 | 3.94 | 6.27 | 3.13 | 72.65 |
| **g31** | 12.82 | 11.25 | 1.23 | 7.08 | 9.97 | 7.68 | 4.60 | 3.62 | 5.91 | 4.61 | 2.07 | 70.83 |
| **g35** | 12.40 | 1.23 | 10.73 | 6.64 | 8.07 | 7.68 | 2.22 | 5.03 | 6.89 | 2.40 | 3.81 | 67.09 |
| **g33** | 11.89 | 6.54 | 1.23 | 7.96 | 9.10 | 7.68 | 4.59 | 4.67 | 3.94 | 5.16 | 3.75 | 66.50 |
| **g1** | 13.93 | 2.40 | 11.20 | 5.92 | 8.47 | 7.68 | 3.34 | 2.27 | 2.95 | 5.72 | 2.28 | 66.15 |
| **g21** | 13.74 | 8.04 | 9.87 | 6.44 | 6.51 | 7.68 | 0.69 | 1.62 | 3.94 | 5.16 | 1.54 | 65.23 |
| **g32** | 13.74 | 6.08 | 1.23 | 8.11 | 8.88 | 7.68 | 3.18 | 2.82 | 3.94 | 5.72 | 2.51 | 63.89 |
| **g20** | 13.56 | 8.63 | 10.04 | 6.40 | 5.08 | 7.28 | 0.61 | 1.66 | 2.95 | 6.27 | 1.00 | 63.48 |
| **g12** | 12.82 | 10.31 | 1.23 | 6.96 | 7.58 | 7.68 | 0.92 | 3.08 | 3.94 | 5.72 | 3.18 | 63.41 |
| **g6** | 13.61 | 10.51 | 1.23 | 6.99 | 5.06 | 6.61 | 0.22 | 1.51 | 5.91 | 6.27 | 3.89 | 61.80 |
| **g18** | 12.44 | 1.23 | 1.23 | 5.90 | 6.34 | 7.68 | 2.42 | 4.02 | 6.89 | 6.27 | 4.07 | 58.49 |
| **g14** | 12.82 | 4.14 | 1.23 | 6.39 | 7.73 | 7.68 | 3.63 | 2.68 | 2.95 | 6.82 | 1.74 | 57.81 |
| **g13** | 13.37 | 5.70 | 1.23 | 6.58 | 7.62 | 7.68 | 1.40 | 2.78 | 3.94 | 5.16 | 2.05 | 57.51 |
| **g26** | 12.44 | 10.25 | 1.23 | 6.76 | 5.50 | 7.12 | 0.22 | 1.12 | 3.94 | 6.27 | 2.01 | 56.86 |
| **g29** | 11.52 | 4.07 | 1.23 | 6.53 | 8.24 | 7.68 | 3.32 | 3.06 | 3.94 | 5.72 | 1.34 | 56.62 |
| **g4** | 12.26 | 1.23 | 1.23 | 6.35 | 9.21 | 7.68 | 4.35 | 4.73 | 3.94 | 2.40 | 2.23 | 55.60 |
| **g24** | 12.63 | 10.98 | 1.23 | 6.74 | 2.42 | 5.72 | 0.00 | 1.11 | 2.95 | 5.72 | 0.53 | 50.03 |
| **g30** | 11.89 | 1.23 | 1.23 | 6.42 | 7.25 | 7.68 | 2.82 | 3.06 | 0.00 | 7.37 | 1.05 | 50.00 |
| **g11** | 11.89 | 3.20 | 1.23 | 6.16 | 5.29 | 7.41 | 0.22 | 1.71 | 2.95 | 5.16 | 0.55 | 45.79 |
| **g15** | 11.33 | 1.23 | 1.23 | 6.17 | 4.89 | 6.58 | 0.19 | 3.19 | 3.94 | 5.72 | 1.07 | 45.53 |
| **g16** | - | 1.23 | 1.23 | 6.26 | 7.59 | 7.68 | 2.92 | 3.91 | 5.91 | 4.61 | 2.23 | 43.56 |
| **g22** | - | 1.23 | 1.23 | 4.57 | 8.53 | 7.68 | 3.57 | 1.92 | 3.94 | 6.82 | 2.87 | 42.37 |
| **g27** | - | 1.23 | 1.23 | 5.12 | 4.62 | 6.34 | 1.72 | 1.87 | 3.94 | 6.27 | 2.95 | 35.31 |
| **g19** | - | 1.23 | 1.23 | 4.96 | 4.64 | 6.00 | 0.23 | 2.60 | 3.94 | 6.27 | 3.77 | 34.89 |
| **g17** | - | 1.23 | 1.23 | 5.12 | 4.72 | 7.68 | 0.10 | 1.96 | 3.94 | 6.27 | 2.03 | 34.28 |
| **g28** | - | 1.23 | 1.23 | 4.70 | 1.43 | 5.49 | 0.06 | 2.10 | 3.94 | 7.37 | 1.80 | 29.36 |
| **g25** | - | 1.23 | 1.23 | 5.51 | 1.86 | 4.48 | 0.06 | 0.66 | 3.94 | 5.16 | 1.65 | 25.80 |
| **g23** | - | 1.23 | 1.23 | 4.82 | 1.48 | 5.42 | 0.06 | 0.69 | 3.94 | 4.61 | 1.12 | 24.59 |

Supplementary Table 11: Results of the non-dimensional and comprehensive scores of different traits for imported potato genotypes in e5.

| **e5** | **The score of 11 components** | | | | | | | | | | | **Total score** |
| --- | --- | --- | --- | --- | --- | --- | --- | --- | --- | --- | --- | --- |
|  | **Quality of french fries** | **Length /width of tubers** | **Dry matter content（%）** | **Length of tubers（cm）** | **The ratio of big tuber** | **The weight per marketable tuber**  **（g / one tuber）** | **Small tuber ratio（width<50mm）** | **Yield（Kg/**  **hm^2^）** | **Maturity** | **Plant vigor** | **Late blight resistance（AUDPC）** |  |
| **g34** | 13.08 | 8.79 | 1.23 | 8.81 | 10.72 | 7.68 | 0.47 | 5.33 | - | - | - | 56.11 |
| **g10** | 13.37 | 11.84 | 9.97 | 7.27 | 2.39 | 4.72 | 0.21 | 2.57 | - | - | - | 52.34 |
| **g3** | 12.26 | 10.33 | 1.23 | 8.40 | 3.59 | 4.95 | 0.35 | 3.34 | - | - | - | 44.47 |
| **g32** | 13.19 | 6.75 | 1.23 | 8.57 | 4.63 | 4.97 | 0.51 | 3.88 | - | - | - | 43.73 |
| **g8** | 13.93 | 11.61 | 1.23 | 6.98 | 0.90 | 4.94 | 0.03 | 1.63 | - | - | - | 41.24 |
| **g1** | 13.93 | 3.25 | 1.23 | 7.50 | 3.61 | 5.27 | 0.34 | 3.10 | - | - | - | 38.25 |
| **g9** | 13.00 | 6.20 | 1.23 | 7.77 | 2.68 | 4.41 | 0.25 | 2.11 | - | - | - | 37.66 |
| **g24** | 13.37 | 11.14 | 1.23 | 6.43 | 0.00 | 3.63 | 0.09 | 1.46 | - | - | - | 37.35 |
| **g33** | 13.19 | 8.29 | 1.23 | 7.11 | 0.93 | 3.54 | 0.30 | 2.67 | - | - | - | 37.25 |
| **g12** | 13.00 | 8.79 | 1.23 | 7.07 | 0.00 | 3.72 | 0.32 | 1.61 | - | - | - | 35.74 |
| **g15** | 11.89 | 1.23 | 10.34 | 4.83 | 0.18 | 3.67 | 0.24 | 2.82 | - | - | - | 35.21 |
| **g35** | 13.14 | 1.23 | 1.23 | 6.47 | 3.02 | 5.22 | 0.38 | 3.03 | - | - | - | 33.73 |
| **g14** | 12.63 | 2.95 | 1.23 | 6.63 | 1.38 | 5.41 | 0.30 | 2.78 | - | - | - | 33.32 |
| **g26** | 12.63 | 9.09 | 1.23 | 5.74 | 0.00 | 3.31 | 0.16 | 0.99 | - | - | - | 33.15 |
| **g20** | 13.56 | 4.13 | 1.23 | 6.22 | 0.44 | 4.52 | 0.19 | 2.05 | - | - | - | 32.35 |
| **g5** | 13.00 | 7.33 | 1.23 | 5.99 | 1.14 | 2.74 | 0.02 | 0.87 | - | - | - | 32.34 |
| **g11** | 13.56 | 1.23 | 1.23 | 6.60 | 2.36 | 4.69 | 0.33 | 2.19 | - | - | - | 32.19 |
| **g29** | 12.26 | 1.23 | 1.23 | 6.89 | 2.31 | 5.01 | 0.24 | 2.18 | - | - | - | 31.34 |
| **g4** | 13.19 | 1.23 | 1.23 | 5.85 | 1.99 | 4.37 | 0.38 | 2.89 | - | - | - | 31.14 |
| **g21** | 14.30 | 1.82 | 1.23 | 6.11 | 0.75 | 4.27 | 0.37 | 2.00 | - | - | - | 30.84 |
| **g7** | 14.12 | 1.23 | 1.23 | 6.25 | 1.47 | 4.46 | 0.14 | 1.45 | - | - | - | 30.36 |
| **g2** | 12.63 | 1.23 | 1.23 | 6.25 | 1.99 | 5.17 | 0.16 | 1.06 | - | - | - | 29.72 |
| **g30** | 14.12 | 1.23 | 1.23 | 5.88 | 0.72 | 3.91 | 0.28 | 2.00 | - | - | - | 29.37 |
| **g13** | 13.56 | 1.23 | 1.23 | 5.69 | 0.00 | 4.53 | 0.23 | 0.69 | - | - | - | 27.16 |
| **g18** | 11.89 | 1.23 | 1.23 | 5.24 | 0.78 | 4.03 | 0.30 | 2.33 | - | - | - | 27.04 |
| **g6** | 12.07 | 1.23 | 1.23 | 5.78 | 0.28 | 3.87 | 0.09 | 2.07 | - | - | - | 26.63 |
| **g25** | 13.37 | 1.23 | 1.23 | 5.48 | 0.00 | 3.87 | 0.20 | 1.19 | - | - | - | 26.57 |
| **g31** | 14.67 | 1.23 | 1.23 | 5.23 | 0.00 | 2.12 | 0.06 | 1.32 | - | - | - | 25.87 |
| **g16** | - | 1.23 | 1.23 | 5.85 | 3.28 | 4.04 | 0.43 | 2.84 | - | - | - | 18.91 |
| **g17** | - | 1.23 | 1.23 | 4.92 | 0.81 | 4.26 | 0.27 | 1.63 | - | - | - | 14.37 |
| **g23** | - | 1.23 | 1.23 | 5.60 | 0.00 | 3.59 | 0.20 | 1.43 | - | - | - | 13.29 |
| **g22** | - | 1.23 | 1.23 | 4.20 | 0.00 | 2.97 | 0.17 | 1.49 | - | - | - | 11.30 |
| **g19** | - | 1.23 | 1.23 | 3.97 | 0.00 | 2.87 | 0.17 | 1.60 | - | - | - | 11.07 |
| **g28** | - | 1.23 | 1.23 | 3.98 | 0.00 | 2.61 | 0.00 | 1.84 | - | - | - | 10.90 |
| **g27** | - | 1.23 | 1.23 | 4.34 | 0.00 | 2.95 | 0.10 | 0.82 | - | - | - | 10.67 |

Note: The values of maturity, plant vigor and AUDPC data of e5 are missing.

Reference：

Kumar, S., Parashar, N., Haleem, A. (2009). Analytical hierarchy process applied to vendor selection problem: small scale, medium scale and large scale industries. Business Intelligence Journal. 2(2), 355–362.

Waxman, A., Stark, J., Thornton, M.K., Olsen, N., Guenthner, J., Novy, R.G. (2019).The Effect of harvest timing on French Fry textural quality of three processing potato varieties: Russet Burbank, Alpine Russet, and Clearwater Russet.American Journal of Potato Research.96(1),33–47.
